# Chromosomal Integration of Huge and Complex *bla*_NDM_-Carrying Genetic Elements in Enterobacteriaceae

**DOI:** 10.3389/fcimb.2021.690799

**Published:** 2021-06-15

**Authors:** Xinhua Luo, Zhe Yin, Lijun Zeng, Lingfei Hu, Xiaoyuan Jiang, Ying Jing, Fangzhou Chen, Dongguo Wang, Yajun Song, Huiying Yang, Dongsheng Zhou

**Affiliations:** ^1^ State Key Laboratory of Pathogen and Biosecurity, Beijing Institute of Microbiology and Epidemiology, Beijing, China; ^2^ The Fifth Medical Center, Chinese Peoples Liberation Army General Hospital, Beijing, China; ^3^ Department of Clinical Laboratory Medicine, Taizhou Municipal Hospital Affiliated With Taizhou University, Taizhou, China

**Keywords:** Enterobacteriaceae, chromosomal integration, *bla*_NDM_, multidrug resistance, mobile genetic elements

## Abstract

In this study, a detailed genetic dissection of the huge and complex *bla*_NDM_-carrying genetic elements and their related mobile genetic elements was performed in Enterobacteriaceae. An extensive comparison was applied to 12 chromosomal genetic elements, including six sequenced in this study and the other six from GenBank. These 12 genetic elements were divided into five groups: a novel IME Tn*6588*; two related IMEs Tn*6523* (SGI1) and Tn*6589*; four related ICEs Tn*6512* (R391), Tn*6575* (ICE*Pvu*ChnBC22), Tn*6576*, and Tn*6577*; Tn*7* and its derivatives Tn*6726* and 40.7-kb Tn*7*-related element; and two related IMEs Tn*6591* (GI*sul2*) and Tn*6590*. At least 51 resistance genes, involved in resistance to 18 different categories of antibiotics and heavy metals, were found in these 12 genetic elements. Notably, Tn*6576* carried another ICE Tn*6582*. In particular, the six *bla*_NDM_-carrying genetic elements Tn*6588*, Tn*6589*, Tn*6575*, Tn*6576*, Tn*6726*, and 40.7-kb Tn*7*-related element contained large accessory multidrug resistance (MDR) regions, each of which had a very complex mosaic structure that comprised intact or residual mobile genetic elements including insertion sequences, unit or composite transposons, integrons, and putative resistance units. Core *bla*_NDM_ genetic environments manifested as four different Tn*125* derivatives and, notably, two or more copies of relevant Tn*125* derivatives were found in each of Tn*6576*, Tn*6588*, Tn*6589*, and 40.7-kb Tn*7*-related element. The huge and complex *bla*_NDM_-carrying genetic elements were assembled from complex transposition and homolog recombination. Firstly identified were eight novel mobile elements, including three ICEs Tn*6576*, Tn*6577*, and Tn*6582*, two IMEs, Tn*6588* and Tn*6589*, two composite transposons Tn*6580a* and Tn*6580b*, and one integron In1718.

## Introduction

New Delhi metallo-β-lactamase (NDM) is able to hydrolyze nearly all β-lactams except aztreonam and thus mediates resistance to penicillins, cephalosporins, and carbapenems (Yong et al., 2009). It is hypothesized that the *bla*_NDM_ gene is originally integrated into the *Acinetobacter* chromosome from an unknown environmental species and then captured by two copies of IS*Aba125*, giving rise to IS*Aba125*-composite transposon Tn*125* (Poirel et al., 2012). With the transposition of Tn*125*, *bla*_NDM_ is disseminated among *Acinetobacter*, Enterobacteriaceae and *Pseudomonas* species; Tn*125* and its *bla*_NDM_-carrying derivatives, with various truncations and deletions, can be found in the accessory resistance regions of bacterial plasmids or chromosomes (Wu et al., 2019). There are reports of chromosomal location of *bla*_NDM_ in Enterobacteriaceae species including *Escherichia coli* (Pfeifer et al., 2011; Poirel et al., 2011; Shen et al., 2017; Reynolds et al., 2019), *Providencia stuartii* (Poirel et al., 2011), *Proteus mirabilis* (Girlich et al., 2015), *Klebsiella pneumoniae* (Sakamoto et al., 2018), and *Proteus vulgaris* (Kong et al., 2020), but few of them are subjective to detailed genetic dissection of *bla*_NDM_-carrying accessory resistance regions (Girlich et al., 2015; Sakamoto et al., 2018; Reynolds et al., 2019; Kong et al., 2020).

Integrative and conjugative elements (ICEs) and integrative and mobilizable elements (IMEs) (Bellanger et al., 2014; Delavat et al., 2017; Botelho and Schulenburg, 2021) are two different types of mobile genetic elements which are frequently integrated into bacterial chromosome, contributing to dissemination of resistance genes. ICEs have the ability to transfer between cells because of their self-encoded conjugation function. It is typically composed of *attL* (attachment site at the left end), *int* (integrase), *xis* (excisionase), *rlx* (relaxase), *oriT* (origin of conjugative replication), *cpl* (coupling protein), a P (TivB)- or F (TivF)-type T4SS machinery (mating pair formation), and *attR* (attachment site at the right end). IMEs are not self-transmissible, and they achieve the intercellular mobility with the help of other conjugative elements that encode proteins involved in complete conjugation function. IMEs typically have *attL*, *int*, *rlx*, *oriT*, and *attR*, but contained no conjugal transfer genes. Tn*7* is a unit transposon with the ability to integrate into bacterial chromosomes and plasmids, and it encodes five core transposition determinants TnsA and TnsB (transposases), TnsC (regulator), and TnsD and TnsE (DNA-binding proteins), as well as three TnsB-binding sites and four TnsB-binding sites at its left and right ends, respectively (Peters, 2014).

In this work, whole-genome sequencing identified four *bla*_NDM-1/-3_-carrying genetic elements plus two additional genetic elements harboring other resistance genes in the chromosomes of four isolates of *Providencia rettgeri*, *Proteus mirabilis*, and *K. pneumoniae.* An extension sequence comparison was then applied to a collection of 12 chromosomal genetic elements (including the above six ones sequenced in this work) that could be grouped into ICEs, IMEs, and Tn*7* unit transposon and its derivatives. Data presented here gave a detailed genetic dissection of the huge and complex *bla*_NDM_-carrying genetic elements and their related mobile genetic elements in multiple Enterobacteriaceae species.

## Materials and Methods

### Bacterial Strains

The four chromosomal *bla*_NDM_-carrying isolates (**Table S1**) were screened from more than two hundred *bla*_NDM_-carrying Enterobacteriaceae isolates routinely collected from China hospitals and livestock farms. *Providencia rettgeri* 1701091 and *Proteus mirabilis* 1701092 (**Table S1**) were recovered in 2017 from the chicken intestinal contents in two different China livestock farms. *K. pneumoniae* QD23 and *Providencia rettgeri* 51003 were recovered from the urine specimens of two different patients with nosocomial infections in two Chinese public hospitals in 2015 and 2017, respectively. Bacterial species identification was performed using genome sequence-based average nucleotide identity analysis (http://www.ezbiocloud.net/tools/ani) (Richter and Rosselló-Móra, 2009).

### Sequencing and Sequence Assembly

Bacterial genomic DNA was isolated using the UltraClean Microbial Kit (Qiagen, NW, Germany) and sequenced from a sheared DNA library with average size of 15 kb (ranged from 10 to 20 kb) on a PacBio RSII sequencer (Pacific Biosciences, CA, USA), as well as a paired-end library with an average insert size of 350 bp (ranged from 150 to 600 kb) on a HiSeq sequencer (Illumina, CA, USA). The paired-end short Illumina reads were used to correct the long PacBio reads utilizing *proovread* (Hackl et al., 2014), and then the corrected PacBio reads were assembled *de novo* utilizing *SMARTdenovo* (https://github.com/ruanjue/smartdenovo).

### Sequence Annotation and Comparison

Open reading frames (ORFs) and pseudogenes were predicted using RAST 2.0 (https://rast.nmpdr.org/) (Brettin et al., 2015) combined with BLASTP/BLASTN searches (Boratyn et al., 2013) against the UniProtKB/Swiss-Prot database (https://web.expasy.org/docs/swiss-prot_guideline.html) (Boutet et al., 2016) and the RefSeq database (https://www.ncbi.nlm.nih.gov/refseq/) (O’Leary et al., 2016). Annotation of resistance genes, mobile elements, and other features were carried out using the online databases including CARD (https://card.mcmaster.ca/browse) (Jia et al., 2017), ResFinder (https://cge.cbs.dtu.dk/services/ResFinder/) (Zankari et al., 2012), ISfinder (https://www-is.biotoul.fr/) (Siguier et al., 2006), INTEGRALL (http://integrall.bio.ua.pt/)? (Moura et al., 2009) and Tn Number Registry (https://www.ucl.ac.uk/eastman/tn-number-registry) (Roberts et al., 2008). Multiple and pairwise sequence comparisons were performed using MUSCLE 3.8.31 (Edgar, 2004) and BLASTN, respectively. Gene organization diagrams were drawn in Inkscape 1.0 (https://inkscape.org/en/). Heatmaps were plotted with MeV 4.9.0 (Saeed et al., 2003).

### Conjugal Transfer

Conjugal transfer experiments were carried out with rifampin-resistant *Escherichia coli* EC600 or sodium azide-resistant *E. coli* J53 being used as a recipient, and the 1701092 or QD23 isolate as a donor. Three milliliters of overnight cultures of each of donor and recipient bacteria were mixed together, harvested and resuspended in 80 μl of Brain Heart Infusion (BHI) broth (BD Biosciences). The mixture was spotted on a 1 cm^2^ hydrophilic nylon membrane filter with a 0.45 µm pore size (Millipore) that was placed on BHI agar (BD Biosciences) plate and then incubated for mating at 37**°**C for 12 to 18 h. Bacteria were washed from filter membrane and spotted on Muller–Hinton (MH) agar (BD Biosciences) plates, for selecting an *E. coli* transconjugant carrying *bla*_NDM_ or carrying *tetA*(C). Then 200 mg/L sodium azide (for J53) or 1,000 mg/L rifampin (for EC600), together with 4 mg/L imipenem (for *bla*_NDM_) or 8 mg/L tetracycline [for *tetA*(C)] was used for transconjugant selection.

### PCR Identification

All the wild-type and transconjugant strains was subjected to PCR amplification followed by amplicon sequencing, for determining the sequences of bacterial 16S rRNA genes (Frank et al., 2008), the presence of key markers such as *bla*_NDM_, *tetA*(C), *int*, and *parM*, and also the location/boundary of mobile genetic elements such as Tn*6588*, Tn*6589*, Tn*6576*, Tn*6577*, and Tn*6590* (data not shown).

### Phenotypic Assays

Activity of Ambler class A/B/D carbapenemases in bacterial cell extracts was determined by a modified CarbaNP test (Feng et al., 2016). Bacterial antimicrobial susceptibility was tested by BioMérieux VITEK 2 and interpreted as per the Clinical and Laboratory Standards Institute (CLSI) guidelines (CLSI, 2020).

### Nucleotide Sequence Accession Numbers

The complete chromosome sequences of the 1701091, 1701092, QD23, and 51003 isolates were submitted to GenBank under accession numbers CP042860, CP042857, CP042858, and CP042861 respectively.

## RESULTS

### Genome Sequencing for Dissection of Chromosomal *bla*_NDM_-Carrying Genetic Elements

The complete genome sequences of four *bla*_NDM_-carrying isolates *Providencia rettgeri* 1701091, *Proteus mirabilis* 1701092, *K. pneumoniae* QD23, and *Providencia rettgeri* 51003 were determined in this work through high-throughput genome sequencing. A total of six chromosome-borne accessory resistance regions were identified: *bla*_NDM-1/-3_-carrying Tn*6588*, Tn*6589*, Tn*6576*, and 40.7-kb Tn*7*-related element from strains 1701091, 1701092, QD23, and 51003, respectively; *tetA*(C)- and *bla*_CTX-M-14_-carrying Tn*6577* were from strain 1701092; and *strAB*-carrying Tn*6590* was from strain 51003.

**Figure 1:**
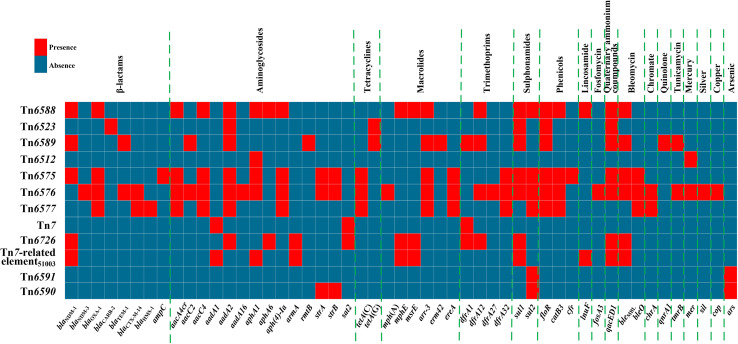
A heatmap of prevalence of resistance genes. The original data are shown in **Table S2**.

A detailed sequence comparison was applied to a collection of 12 chromosomal genetic elements, which included the above mentioned six genetic elements sequenced in this study, together with six additional ones from GenBank (four reference/prototype ones Tn*6523*, Tn*6512*, Tn*7*, and Tn*6591*, and two *bla*_NDM_-carrying ones Tn*6575* and Tn*6726*). These 12 genetic elements could be further divided into five distinct groups: a novel IME Tn*6588*; two related IMEs Tn*6523* and Tn*6589*; four related ICEs Tn*6512*, Tn*6575*, Tn*6576* and Tn*6577*; Tn*7* and its two derivatives Tn*6726* and 40.7-kb Tn*7*-related element; and two related IMEs Tn*6591* and Tn*6590* (**Table 1**). Six (Tn*6588*, Tn*6589*, Tn*6575*, Tn*6576*, Tn*6726*, and 40.7-kb Tn*7*-related element) of them harbored *bla*_NDM_. At least 51 resistance genes, involved in resistance to 18 different categories of antibiotics and heavy metals, were identified in these 12 elements (**Figure 1** and **Table S2**).

**Table 1:** Major features of genetic elements characterized in this work.

Group	Genetic element	Accession number	Presence (+)or absence (−) of *bla*_NDM_	Chromosomal nucleotide position	Length(bp)	Host bacterium	Reference
Novel IME	Tn*6588*	CP042860	+	4048158.4148181	100,024	*Providencia rettgeri* 1701091	This study
Tn*6523*-related IMEs	Tn*6523*	AF261825	**−**	Not applicable	42,451	*Salmonella enterica* Typhimurium DT104	(Boyd et al., 2000)
	Tn*6589*	CP042857	+	4033353.4127100	93,748	*Proteus mirabilis* 1701092	This study
Tn*6512*-related ICEs	Tn*6512*	AY090559	**−**	Not applicable	88,549	*Providencia rettgeri* 107	(Boltner et al., 2002)
	Tn*6575*	MH160822	+	Not applicable	146,895	*Proteus vulgaris* BC22	(Kong et al., 2020)
	Tn*6576*	CP042858	+	4485620.5019721	534,102	*K. pneumoniae* D23	This study
	Tn*6577*	CP042857	**−**	3239622.3377168	137,547	*Proteus mirabilis* 1701092	This study
Tn*7*-related elements	Tn*7*	KX117211	**−**	Not applicable	14,067	*E. coli* 3.5-R3	(Peters and Craig, 2001)
	Tn*6726*	AP018750	+	5052592.5228430	175,839	*K. pneumoniae * KP64	(Sakamoto et al., 2018)
	40.7-kb Tn*7*-related element	CP042861	+	31628.72403	40,776	*Providencia rettgeri* 51003	This study
Tn*6591*-related IMEs	Tn*6591*	AE014073	**−**	2598547.2614010	15,464	*Shigella flexneri* 2457T	(Boyd et al., 2000)
	Tn*6590*	CP042861	**−**	2252695.2268315	15,621	*Providencia rettgeri* 51003	This study

For each group, all the fully sequenced and non-redundant bla_NDM_-carrying genetic elements available in GenBank (last accessed 15 December 2019) are included.

## A Novel IME Tn*6588*

**Figure 2:**
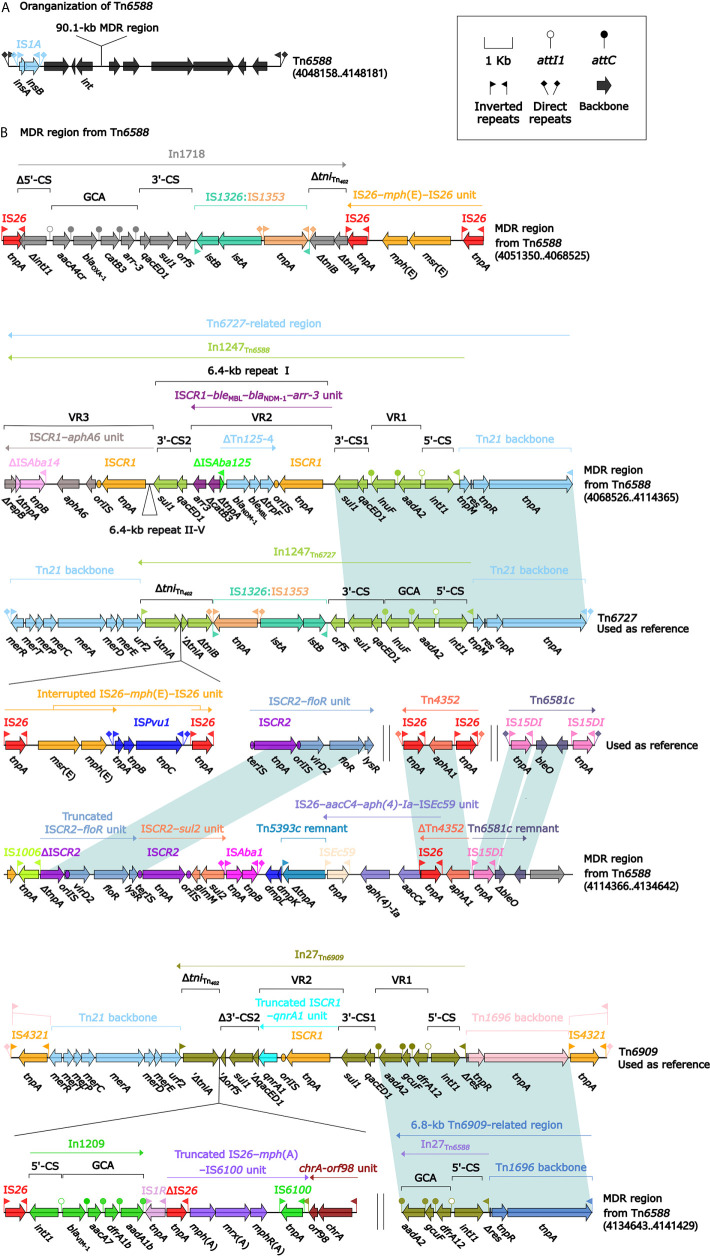
Shown are the organization of Tn*6588* **(A)**, and MDR region from Tn*6588* **(B)**. Genes are denoted by arrows. Genes, mobile elements, and other features are colored based on their functional classification. Shading denotes regions of homology (nucleotide identity ≥95%). Numbers in brackets indicate nucleotide positions within the chromosome of strain 1701091. The accession numbers of Tn*6727*, IS*CR2*–*floR* unit, Tn*4352* (Wrighton and Strike, 1987), Tn*6581c*, and Tn*6909* used as reference are CP047346, CP042857, CP042858, CP042857, and CP032168, respectively.

Tn*6588* (100.0 kb in length) was inserted into the chromosomal *orf1407* gene (cytochrome c551 peroxidase). Tn*6588* had a 9.2-kb backbone (containing *int*) with insertion of two accessory modules: IS*1A* and a 90.1-kb multidrug resistance (MDR) region, and it had terminal 35-bp *attL*/*attR* pairs and were further bracketed by 5-bp direct repeats (DRs; target site duplication signals for transposition) (**Figure 2A**). The MDR region contained a total of 19 resistance genes including *bla*_NDM-1_ (**Figure 1** and **Table S2**), which were located at eight different resistance loci: In1718, IS*26*–*mph*(E)–IS*26* unit, In1247, a truncated IS*CR2*–*floR* unit, IS*CR2*–*sul2* unit, IS*Ec59*–*aph(4)-Ia*–*aacC4*–IS*26* unit, ΔTn*4352* containing *aphA1*, and a 6.8-kb In27-carrying Tn*6909*-related region (**Figure 2B**).

In1718 was a concise class 1 integron with a gene cassette array (GCA) *aacA4cr*–*bla*_OXA-1_–*catB3*–*arr-3*. In1247 was a complex class 1 integron containing VR1 (variable region 1=GCA: *aadA2*–*lnuF*), five copies of 6.4-kb repeat [VR2 (IS*CR1*–*ble*_MBL_–*bla*_NDM-1_–*arr-3* unit) plus 3′-CS2 (a second 3′-conseved segment)], and VR3 (IS*CR1*–*aphA6* unit). In1247 plus a Tn*21* core transposition module *tnpAR*–*res*–*tnpM* in this MDR region were genetically related to the Tn*3*-family unit transposon Tn*6727* (Partridge et al., 2001), which was initially found in *Proteus vulgaris* and originally associated with Tn*21*. The 6.8-kb Tn*6909*-related region looked like a truncated version of Tn*3*-family unit transposon Tn*6909* that was originally associated with Tn*1696* and Tn*21* (Partridge et al., 2001).

### Two Related IMEs Tn*6523* and Tn*6589*

**Figure 3:**
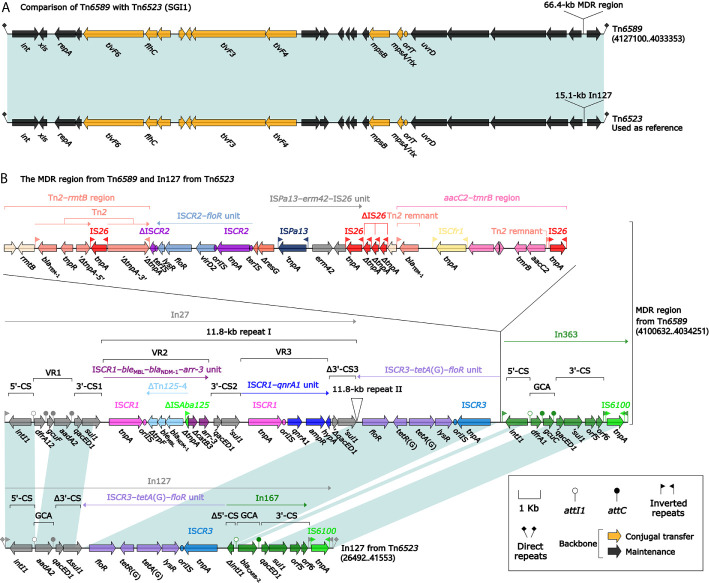
Shown are two Tn*6523*-related transposons **(A)**, and MDR region from Tn*6589* and In127 from Tn*6523* **(B)**. Genes are denoted by arrows. Genes, mobile elements, and other features are colored based on their functional classification. Shading denotes regions of homology (nucleotide identity ≥95%). Numbers in brackets indicate nucleotide positions within Tn*6523* (Boyd et al., 2000) and the chromosome of strain 1701092. The accession number of Tn*6523* used as reference is AF261825.

Tn*6523*, a 42.4-kb IME initially found in *Salmonella enterica* serovar Typhimurium DT104 (Boyd et al., 2000), had a 27.2-kb backbone (containing *attL*, *int*, *xis*, *rlx*, *oriT*, and *attR*) with insertion of a single accessory module In127. Besides the GCA (*aadA2*), In127 captured additional two resistance loci: IS*CR3*–*tetA*(G)–*floR* unit and a *bla*_CARB-2_-carrying In167. The backbone of Tn*6589* was almost identical to Tn*6523* but integrated with a 66.4-kb MDR region instead of In127 (**Figure 3A**). This MDR region contained a total of 16 resistance genes including *bla*_NDM-1_ (**Figure 1** and **Table S2**), which were located at seven different resistance loci: In27, IS*CR3*–*tetA*(G)–*floR* unit, Tn*2*–*rmtB* region, IS*CR2*–*floR* unit, IS*Pa13*–*erm42*–IS*26* unit, *aacC2*–*tmrB* region, and In363 with a GCA *dfrA1*–*gcuC* (**Figure 3B**). In27 in this MDR region was a complex class 1 integron, which carried VR1 (GCA: *dfrA12*–*gcuF*–*aadA2*), and two copies of 11.8-kb repeat region [VR2 (IS*CR1*–*ble*_MBL_–*bla*_NDM-1_–*arr-3* unit) + 3′-CS2 + VR3 (IS*CR1*–*qnrA1* unit) + 3′-CS3] (**Figure 3B**).

### Four Related ICEs Tn*6512*, Tn*6575*, Tn*6576*, and Tn*6577*

Tn*6512*, an 88.5-kb ICE initially found in *Providencia rettgeri* 107 (Boltner et al., 2002), was composed of an 82.4-kb backbone with insertion of three accessory modules: IS*15DI*-composite transposon Tn*6578* (containing *aphA1*), IS*Prre1* and IS*Prre2* (**Figure S1**). Tn*6512*, Tn*6575* (146.9 kb in length) (Kong et al., 2020), Tn*6576* (534.1 kb in length), and Tn*6577* (137.5 kb in length) had similar backbones and, especially, shared the core backbone genes *attL*, *int*, *xis*, *rlx*, *ori*, *cpl*, a TivF-type T4SS gene set, and *attR* (**Figure S1**). All these four ICEs were integrated at the same site within the chromosomal gene *prfC* (peptide chain release factor 3).

Each of Tn*6575*, Tn*6576*, and Tn*6577* had two accessory modules. Firstly, a 85.0-kb MDR region, a novel 406.4-kb ICE Tn*6582*, and a 11.1-kb *bla*_HMS_-*sul2* region were inserted at the same site within *umuC* of Tn*6575*, Tn*6576*, and Tn*6577*, respectively, which led to different truncations of surrounding backbone regions of Tn*6575* and Tn*6577* but not Tn*6576*. Secondly, IS*Ppu12*, Tn*6580b* (55.2 kb in length), and Tn*6580a* (50.0 kb in length) were integrated at the same site downstream of *orf714* of Tn*6575*, Tn*6576*, and Tn*6577*, respectively (**Figure S1**).

**Figure 4:**
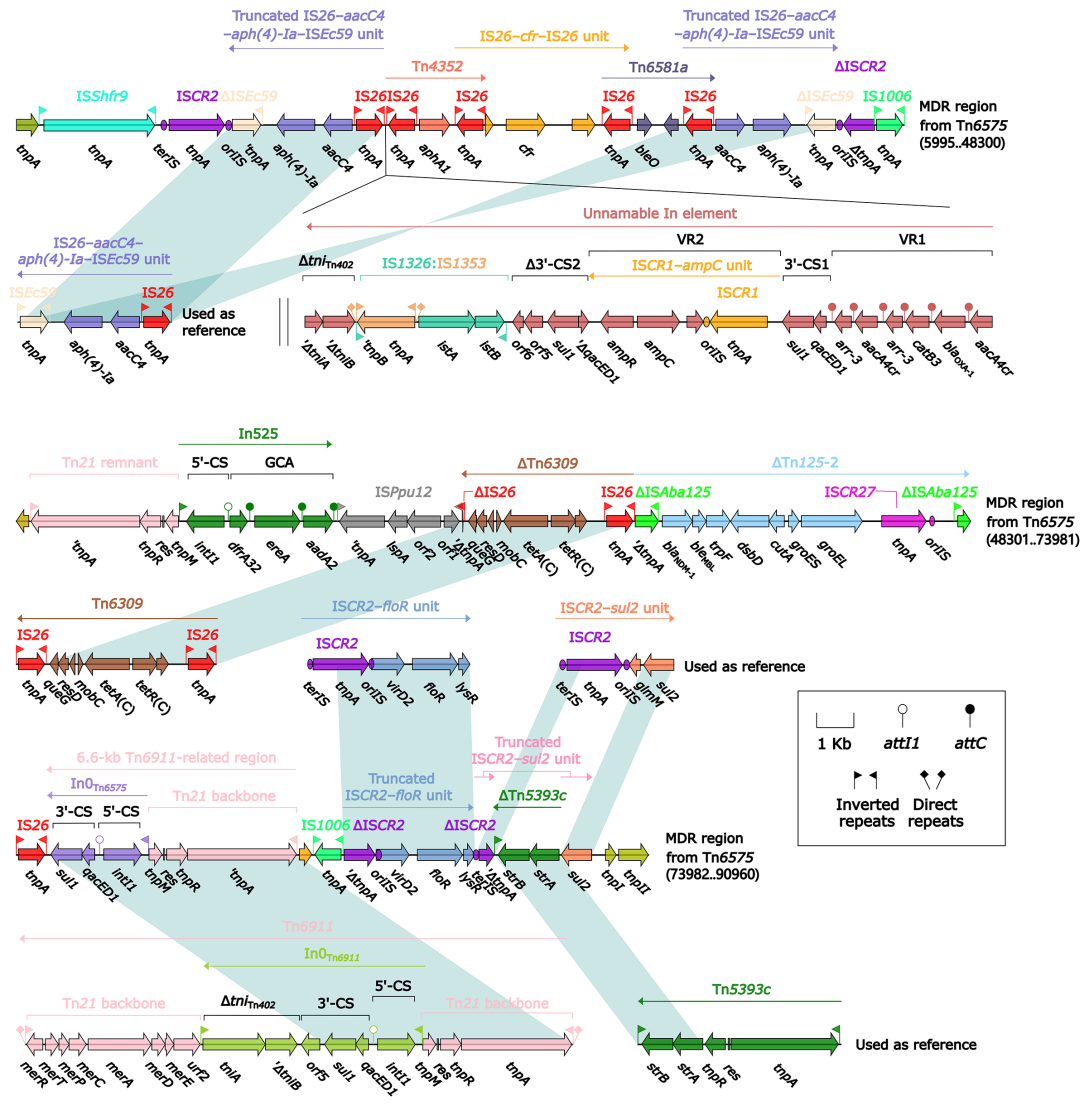
Organization of MDR region from Tn*6575*, and comparison to related regions. Genes are denoted by arrows. Genes, mobile elements, and other features are colored based on their functional classification. Shading denotes regions of homology (nucleotide identity ≥95%). Numbers in brackets indicate nucleotide positions within Tn*6575*. The accession numbers of IS*26*–*aacC4*–*aph(4)-Ia*–IS*Ec59* unit, Tn*6309* (Sun et al., 2016), Tn*6911*, Tn*5393c* (L’Abée-Lund and Sørum, 2000), IS*CR2*–*floR* unit, IS*CR2*–*sul2* unit used as reference are CP042857, KX710094, KT033469, AF262622, CP042857, and AE014073, respectively.

The 85.0-kb MDR region of Tn*6575* contained a total of 22 resistance genes including *bla*_NDM-1_ (**Figure 1** and **Table S2**), which were located at 12 different resistance loci: two copies of a truncated IS*Ec59*–*aph(4)-Ia*–*aacC4*–IS*26* unit, an unnamable In element (harboring a long GCA *aacA4cr*–*bla*_OXA-1_–*catB3*–*arr-3*–*aacA4cr*–*arr-3* but lacking the whole 5′-CS), *aphA1*-containing Tn*4352*, IS*26*–*cfr*–IS*26* unit, IS*26*-composite transposon Tn*6581a* containing *bleO*, In525 (GCA: *dfrA32*–*ereA*–*aadA2*), *tetA*(C)-containing ΔTn*6309*, *bla*_NDM-1_-containing ΔTn*125*-2, a 6.6-kb In0-carrying Tn*6911*-related region, a truncated IS*CR2*–*floR* unit, a truncated IS*CR2*–*sul2* unit, and *strAB*-containing ΔTn*5393c* (**Figure 4**). The Tn*6911*-related region in this MDR region looked like a truncated version of Tn*3*-family unit transposon Tn*6911* that was originally associated with Tn*21* (Partridge et al., 2001).

**Figure 5:**
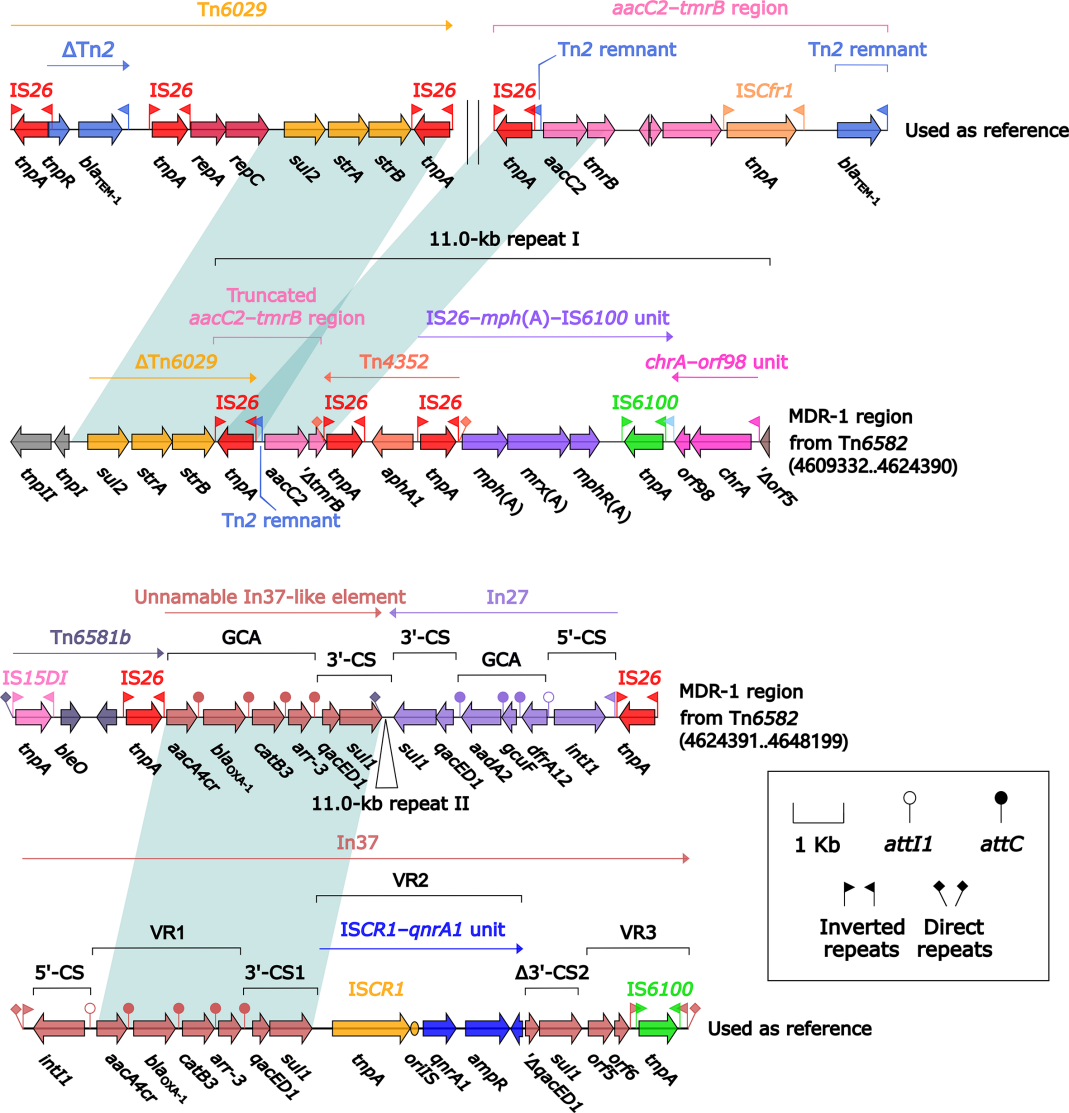
Organization of MDR-1 region from Tn*6582*, and comparison to related regions. Genes are denoted by arrows. Genes, mobile elements, and other features are colored based on their functional classification. Shading denotes regions of homology (nucleotide identity ≥95%). Numbers in brackets indicate nucleotide positions within the chromosome of strain QD23. The accession numbers of Tn*6029* (Cain et al., 2010), *aacC2*–*tmrB* region (Partridge et al., 2012), and In37 (Wang et al., 2003) used as reference are GQ150541, JX101693, and AY259086, respectively.

**Figure 6:**
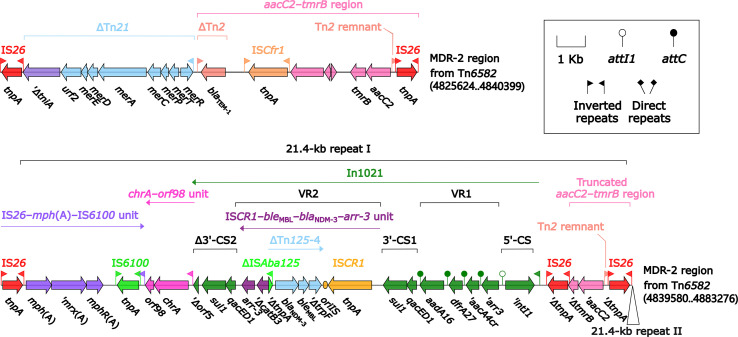
Organization of MDR-2 region from Tn*6582*. Genes are denoted by arrows. Genes, mobile elements, and other features are colored based on their functional classification. Shading denotes regions of homology (nucleotide identity ≥95%). Numbers in brackets indicate nucleotide positions within the chromosome of strain QD23.

Tn*6582* had a complete set of core ICE backbone determinants and, moreover, a lot of accessory modules: two MDR (MDR-1 and MDR-2) regions, *sil*–*cop* region as found in IncHI2 plasmid R478 (Gilmour et al., 2004), *floR*–*strAB*–*sul2* region (containing ΔTn*5393c* and a truncated IS*CR2*–*floR* unit), and multiple intact or residue IS elements (**Figure S2**). The 38.9-kb MDR-1 region (**Figure 5**) comprised ΔTn*6029* (containing *sul2* and *strAB*), Tn*6581b*, an unnamable In37-like element (harboring the In37 GCA *aacA4cr*–*bla*_OXA-1_–*catB3*–*arr-3* but lack of the whole 5′-CS), In27 (GCA: *dfrA12*–*gcuF*–*aadA2*), and two copies of 11.0-kb repeat (each copy harbored a truncated *aacC2*–*tmrB* region, Tn*4352*, IS*26*–*mph*(A)–IS*6100* unit, and *chrA*-*orf98* unit). The 43.7-kb MDR-2 region (**Figure 6**) comprised ΔTn*21* (containing *mer*), *aacC2*–*tmrB* region, and two copies of 21.4-kb repeat (each copy harbored IS*26*–*mph*(A)–IS*6100* unit, *chrA*-*orf98* unit, In1021, and a truncated *aacC2*–*tmrB* region). In1021 was a complex class 1 integron containing VR1 (GCA: *aacA4cr*–*arr3*–*dfrA27*–*aadA16*) and VR2 (IS*CR1*–*ble*_MBL_–*bla*_NDM-3_–*arr-3* unit).

**Figure 7:**
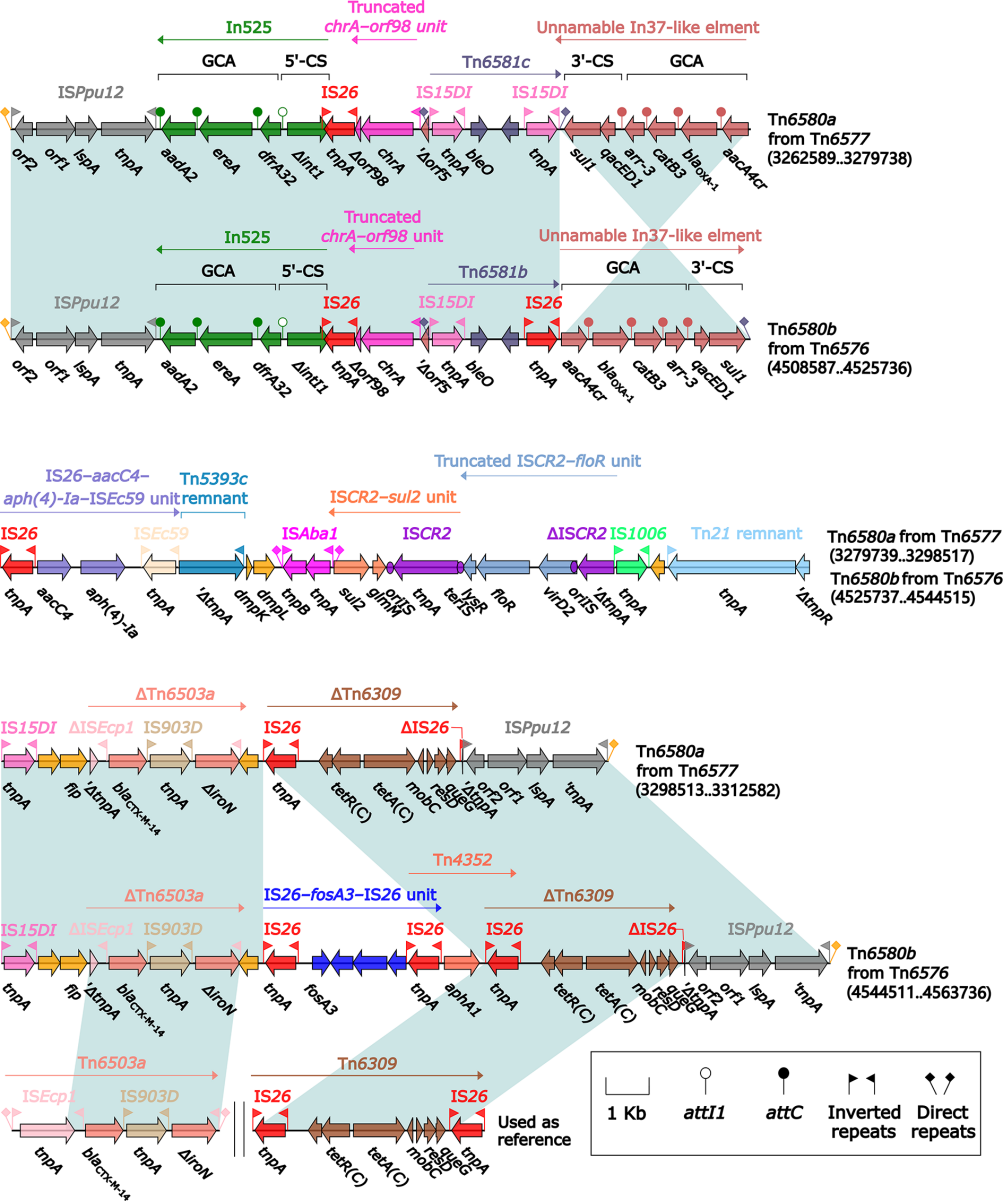
Comparison of Tn*6580a* from Tn*6577* and Tn*6580b* from Tn*6576*, and related regions. Genes are denoted by arrows. Genes, mobile elements, and other features are colored based on their functional classification. Shading denotes regions of homology (nucleotide identity ≥95%). Numbers in brackets indicate nucleotide positions within the chromosomes of strains 1701092 and QD23. The accession numbers of Tn*6503a* (Feng et al., 2015) and Tn*6309* (Sun et al., 2016) used as reference are KP987215 and KX710094, respectively.

The IS*Ppu12*-composite transposon Tn*6580a* contained a total of 17 resistance genes (**Figure 1** and **Table S2**), which were located at nine different resistance loci: In525 (GCA: *dfrA32*–*ereA*–*aadA2*), a truncated *chrA*–*orf98* unit, Tn*6581c*, the unnamable In37-like element as described above, IS*Ec59*–*aph(4)-Ia*–*aacC4*–IS*26* unit, IS*CR2*–*sul2* unit, IS*CR2*–*floR* unit, *bla*_CTX-M-14_-containing ΔTn*6503a*, and *tetA*(C)-containing ΔTn*6309* (**Figure 7**). Three major modular differences were recognized in Tn*6580b* relative to Tn*6580a*: i) replacement of Tn*6581c* by Tn*6581b*; ii) inversion of the unnamable In37-like element; and iii) insertion of IS*26*–*fosA3*–IS*26* unit together with Tn*4352* at a site between ΔTn*6503a* and ΔTn*6309*.

### Three Related Unit Transposons Tn*7*, Tn*6726*, and the 40.7-kb Tn*7*-Related Element

**Figure 8:**
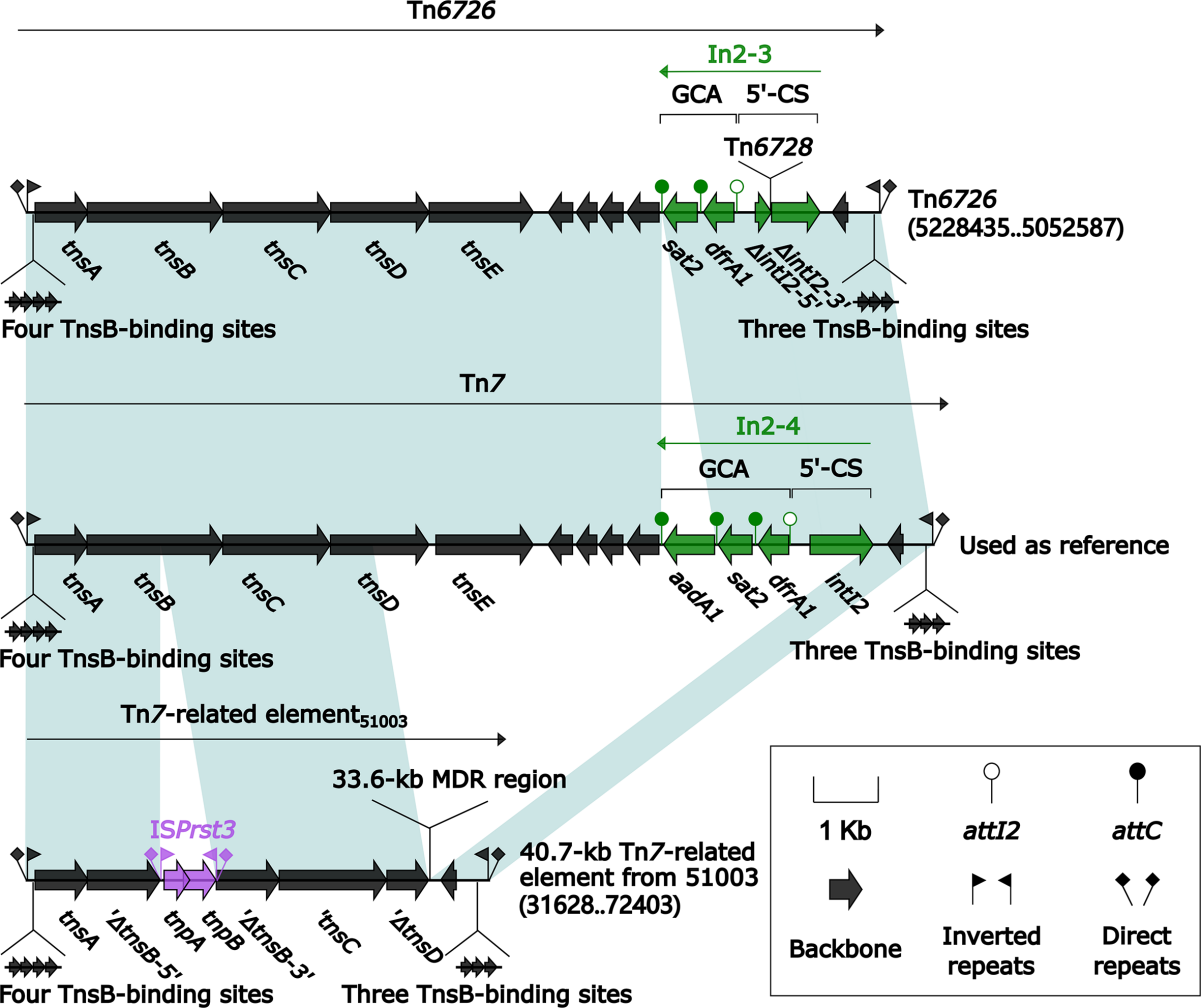
Comparison of Tn*7* and its two derivatives Tn*6726* and 40.7-kb Tn*7*-related element. Genes are denoted by arrows. Genes, mobile elements, and other features are colored based on their functional classification. Shading denotes regions of homology (nucleotide identity ≥95%). Numbers in brackets indicate nucleotide positions within the chromosomes of strains KP64 and 51003. The accession number of Tn*7* used as reference is KX117211.

**Figure 9:**
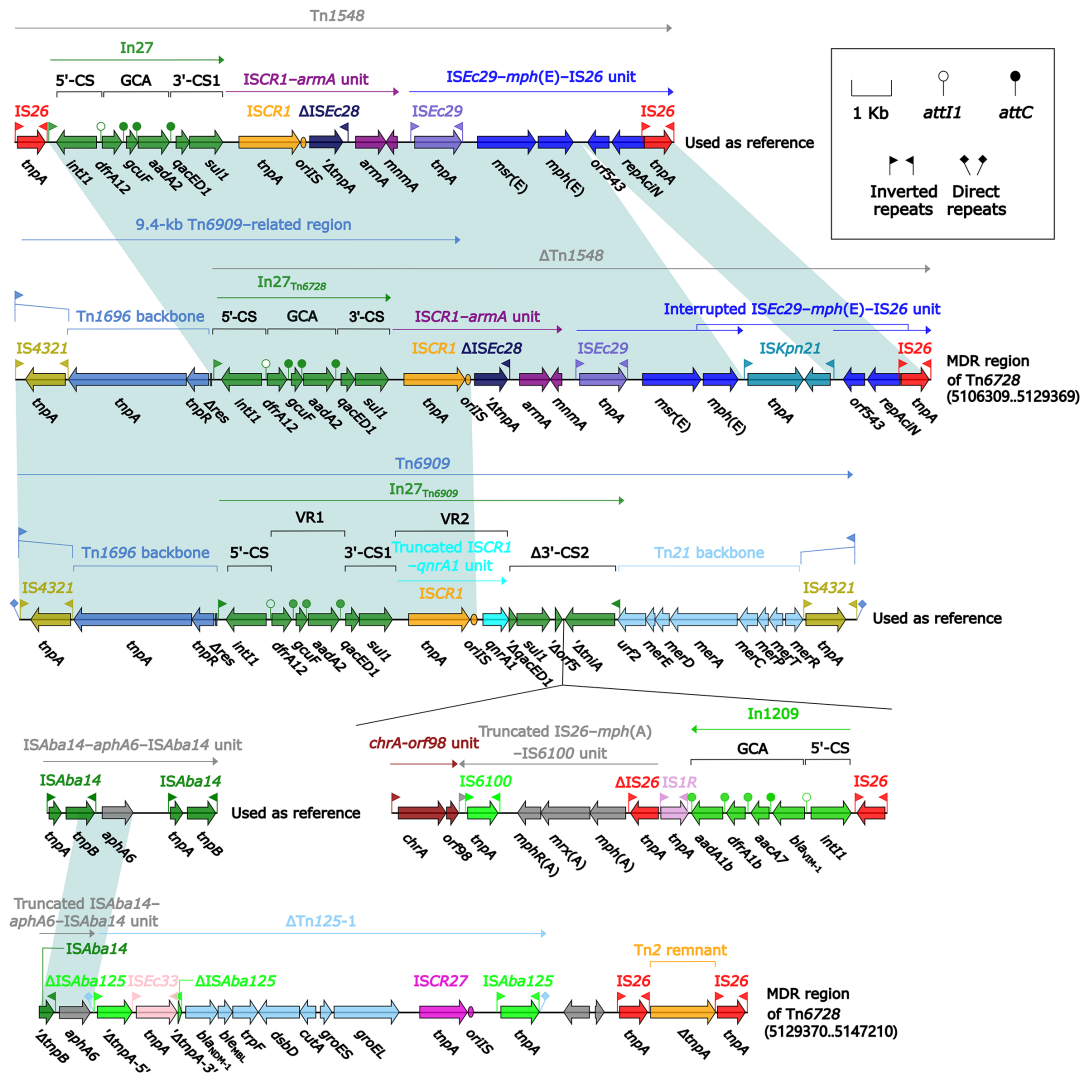
Organization of MDR region from Tn*6728*, and comparison to related regions. Genes are denoted by arrows. Genes, mobile elements, and other features are colored based on their functional classification. Shading denotes regions of homology (nucleotide identity ≥95%). Numbers in brackets indicate nucleotide positions within the chromosome of strain KP64. The accession numbers of Tn*1548* (Galimand et al., 2005), Tn*6909*, and IS*Aba14*–*aphA6*–IS*Aba14* unit used as reference are AF550415, CP032168, and CP046406, respectively.

All of Tn*7* (14.1 kb in length), Tn*6726* (175.8 kb in length) (Sakamoto et al., 2018), and 40.7-kb Tn*7*-related element were integrated at a site downstream of the chromosomal gene *glmS* (glutamine-fructose-6-phosphate aminotransferase). Tn*6726* differed from Tn*7* by acquisition of In2-3 (GCA: dfrA1) instead of In2-4 (GCA: *dfrA1*–*aadA1*) and, moreover, a 162.6-kb IS*Kpn26*-composite transposon Tn*6728* was inserted at a site within *intI2* of In2-3 (**Figure 8**). Tn*6728* harbored a 40.9-kb MDR region as well as an array of IncHI3 core backbone genes (**Figure S3**). This 40.9-kb MDR region included a 9.4-kb Tn*6909*-related region together with ΔTn*1548* (these two shared In27), a truncated IS*Aba14*–*aphA6*–IS*Aba14* unit, and *bla*_NDM-1_-carrying ΔTn*125*-1 (**Figure 9**).

**Figure 10:**
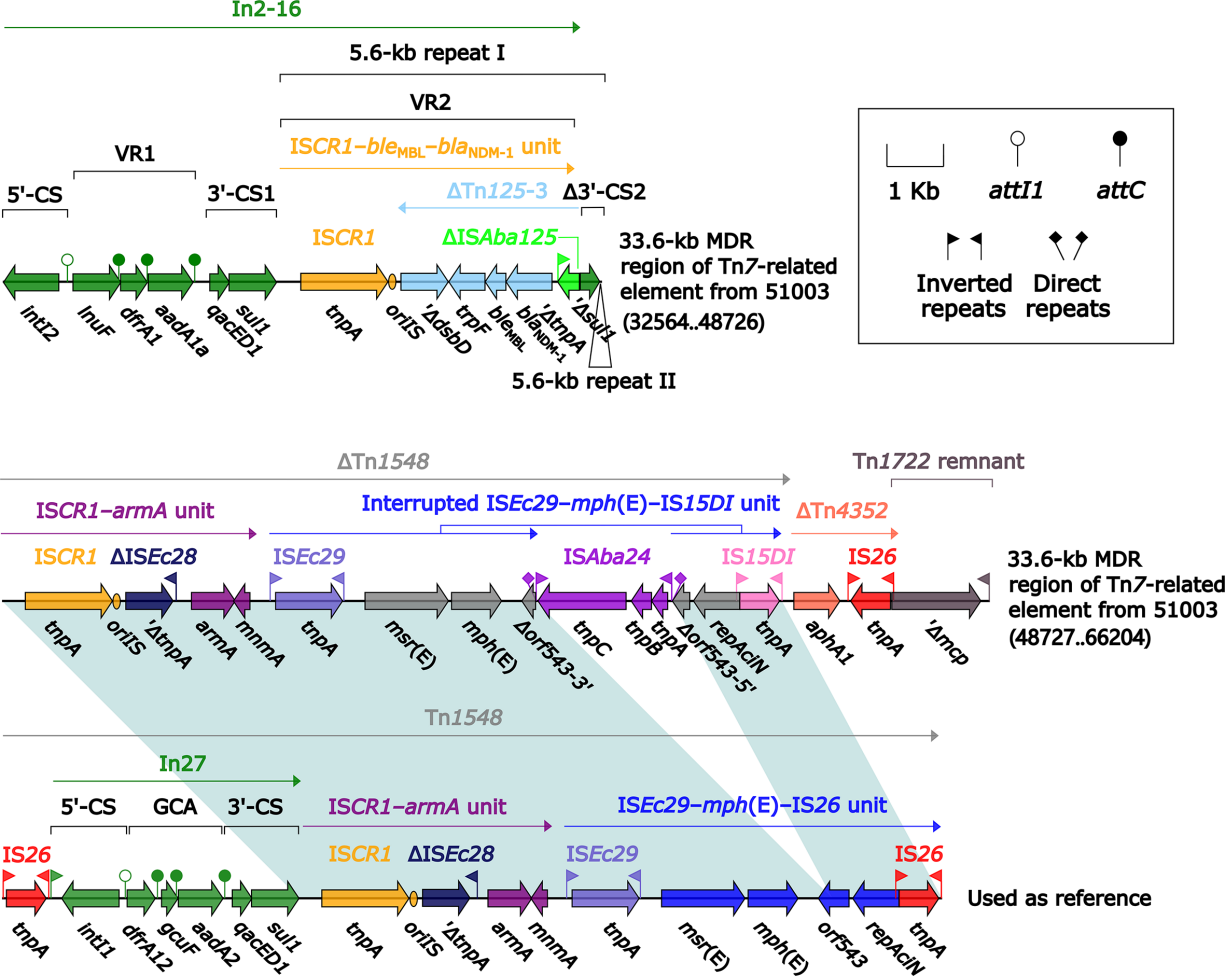
Organization of MDR region from 40.7-kb Tn*7*-related element, and comparison to related regions. Genes are denoted by arrows. Genes, mobile elements, and other features are colored based on their functional classification. Shading denotes regions of homology (nucleotide identity ≥95%). Numbers in brackets indicate nucleotide positions within the chromosome of strain 51003. The accession number of Tn*1548* (Galimand et al., 2005) used as reference is AF550415.

Compared with Tn*7*, 40.7-kb Tn*7*-related element underwent two insertion events: i) *tnsABCDE* was truncated by insertion of a 33.6-kb MDR region; and ii) *tnsB* was interrupted by insertion of IS*Prst3*; this element could not be discriminated as an intact Tn*7*-like transposon due to the presence of an incomplete *tnsABCDE* module (**Figure 8**). This 33.6-kb MDR region contained In2-16, ΔTn*1548*, and *aphA1*-carrying ΔTn*4352.* In2-16 carried VR1 (GCA: *lnu*(F)–*dfrA1*–*aadA1a*) and two copies of 5.6-kb repeat [VR2 (IS*CR1*–*ble*_MBL_–*bla*_NDM-1_ unit) plus Δ3′-CS2] (**Figure 10**).

IS*Ec29*–*mph*(E)–*IS26*/IS*15DI* unit and IS*CR1*–*armA* unit were presented in both ΔTn*1548* from Tn*6728* and that from 40.7-kb Tn*7*-related element, whereas In27 was found in the former ΔTn*1548* rather than the later one; in addition, IS*Ec29*–*mph*(E)–*IS26*/IS*15DI* unit was interrupted by insertion of two different IS elements IS*Kpn21* and IS*Aba24*, respectively, in these two ΔTn*1548* (**Figures 9** and **10**).

### Two Related IMEs Tn*6591* and Tn*6590*

**Figure 11:**
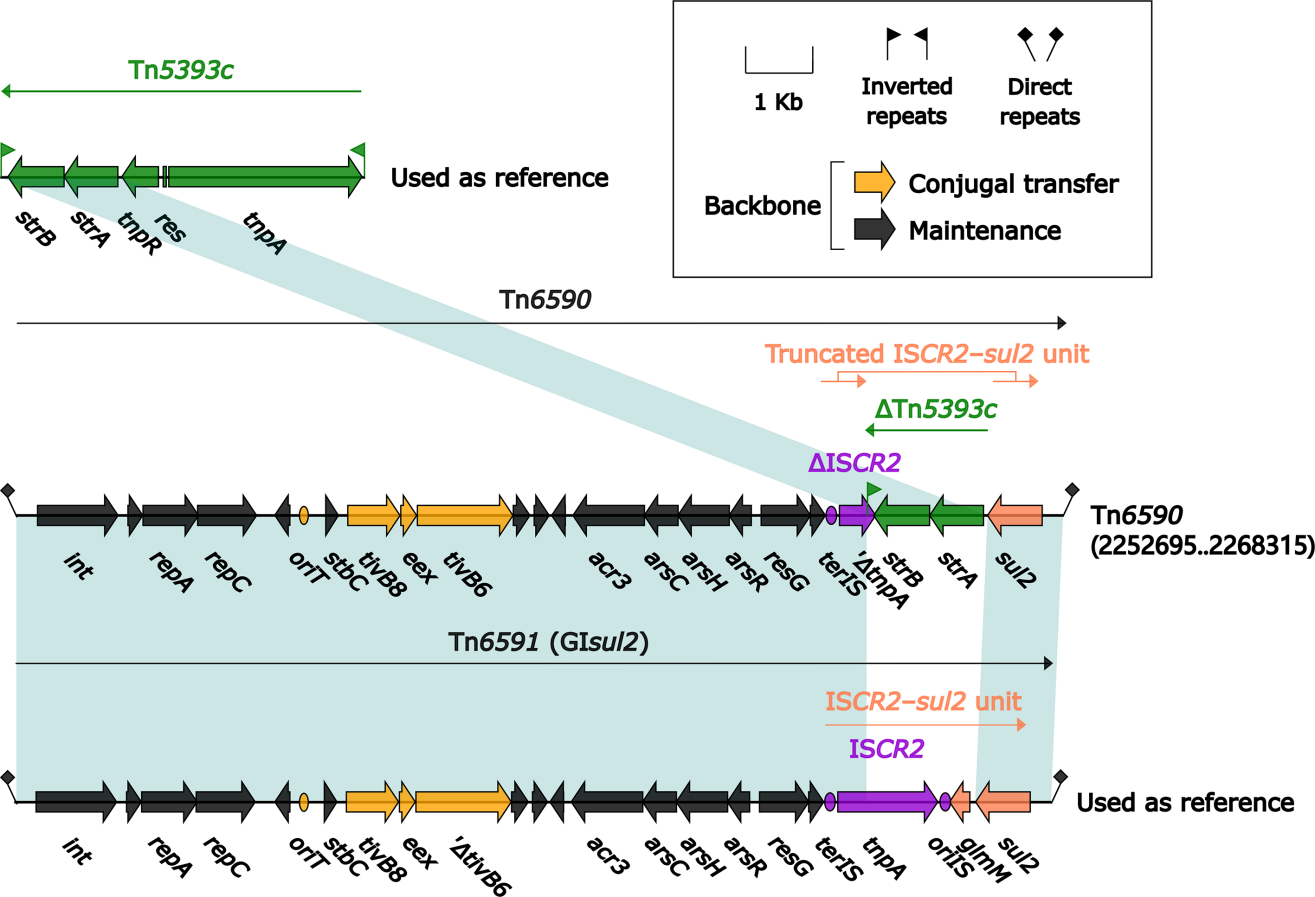
Organization of two related IMEs Tn*6590* and Tn*6591*, and comparison to related regions. Genes are denoted by arrows. Genes, mobile elements, and other features are colored based on their functional classification. Shading denotes regions of homology (nucleotide identity ≥95%). Numbers in brackets indicate nucleotide positions within the chromosome of strain 51003. The accession number of Tn*5393c* (L’Abée-Lund and Sørum, 2000) used as reference is AF262622.

The 15.5-kb IME Tn*6591* (GI*sul2*) (Wei et al., 2003), initially found in *Shigella flexneri* 2457T, was integrated into the chromosomal gene *guaA* (glutamine-hydrolyzing GMP synthase) and had a 12.6-kb backbone (containing *attL*, *int*, *oriT* and *attR*) with insertion of a single accessory module IS*CR2*-*sul2* unit (**Figure 11**). Tn*6590* (15.6 kb in length) was integrated at the same chromosomal site, and Tn*6590* differed from Tn*6591* by only truncation of IS*CR2*–*sul2* unit due to insertion of *strAB*-carrying ΔTn*5393c*.

### Plasmids of the Four Strains Sequenced in This Study

*Proteus mirabilis* 1701092 carried no plasmids, and all accessory resistance regions (Tn*6577* and Tn*6589*) were located in the chromosome. Besides chromosome-borne accessory resistance regions, an IncFII plasmid p701091-FII (carrying no resistance genes), an IncI plasmid pQD23-CTXM [harboring *bla*_CTX-M-104_ and *erm*(B)], and an IncFII plasmid p51003-FII (containing *bla*_TEM-1B_ and *bla*_CTX-M-3_) together with an Col3M plasmid p51003-qnrD (having *qnrD*) were identified in *Providencia* spp. 1701091, *K. pneumoniae* QD23, and *Providencia* spp. 51003, respectively. Coexistance of a large array of resistance genes in both chromosome and plasmids of a single bacterial isolate makes it tends to become extensively resistant.

### Transferability and Antimicrobial Susceptibility

This work identified three ICEs Tn*6577*, Tn*6582* and Tn*6576* in total, all of which had essential conjugal transfer genes. Notably, Tn*6582* was located within Tn*6576*. As for conjugation experiments, Tn*6577* was transferred from the wild-type isolate (susceptible to rifampin) into *E. coli* EC600, generating the transconjugant Tn*6577*–TETA(C)–EC600; Tn*6582* could be transferred from the wild-type isolate (non-susceptible to rifampin but susceptible to sodium azide) in *E. coli* J53 to obtain Tn*6582*–NDM–J53, but repeated conjugation attempts failed to transfer Tn*6576* into *E. coli* J53. Tn*6577*–TETA(C)–EC600 was highly resistant to tetracycline and ceftriaxone owing to presence of *tetA*(C) and *bla*_CTX-M-14_. Tn*6582*–NDM–J53 was highly resistant to ceftriaxone and imipenem resulted from production of NDM enzyme (data not shown). The Ambler class B carbapenemase activity was detected in Tn*6582*–NDM–J53 and its wild-type isolate.

## Discussion

Since the *bla*_NDM_ gene was initially identified in India in 2009 (Yong et al., 2009), it spread rapidly all over the world (Dortet et al., 2014). Although *bla*_NDM_ was initially discovered in a plasmid of *K. pneumoniae* (Yong et al., 2009), the chromosomal location of *bla*_NDM_ in Enterobacteriaceae species has been reported in recent years (Pfeifer et al., 2011; Poirel et al., 2011; Girlich et al., 2015; Shen et al., 2017; Sakamoto et al., 2018; Reynolds et al., 2019; Kong et al., 2020). There were few reports related to a detailed genetic dissection of different kinds of *bla*_NDM_-carrying accessory resistance regions in the chromosomes (Girlich et al., 2015; Sakamoto et al., 2018; Reynolds et al., 2019; Kong et al., 2020), but none of them had a systematic summary for these *bla*_NDM_-carrying mobile genetic elements.

Data presented here involved a total of six chromosomal *bla*_NDM_ *-*carrying genetic elements Tn*6575*, Tn*6726*, Tn*6588*, Tn*6589*, Tn*6576*, and 40.7-kb Tn*7*-related element, and the last four were sequenced in this work. These six genetic elements belonged to three different categories: ICEs (Tn*6575* and Tn*6576*), IMEs (Tn*6588* and Tn*6589*), and two derivatives (Tn*6726* and 40.7-kb Tn*7*-related element) of Tn*7* unit transposon. Notably, Tn*6576* carried another ICE Tn*6582*. These ICEs and IMEs would have the intercellular self-mobility as they carried essential conjugal transfer genes (Bellanger et al., 2014; Botelho and Schulenburg, 2021). Tn*6726* would have the intracellular mobility as it had a complete core transposition module *tnsABCDE*, while 40.7-kb Tn*7*-related element would loss its mobility due to lesion in *tnsABCDE*.

Tn*6512*-related ICEs were frequently reported in *Vibrio*, *Proteus*, and *Shewanella* (Burrus et al., 2006; Nonaka et al., 2012; Lei et al., 2016; Fang et al., 2018). Tn*6575* and Tn*6576* were the only two *bla*_NDM_-carrying Tn*6512*-related ICEs (last accessed 15 December 2019). Tn*6523*-related IMEs were frequently reported in *Salmonella* and *Proteus mirabilis* (Hall, 2010; Siebor and Neuwirth, 2013; Sung et al., 2017). This study presented Tn*6589*, the first *bla*_NDM_-carrying Tn*6523*-related IME. Tn*7*, and its derivatives had the ability to integrate into bacterial plasmids and chromosomes (Peters, 2014). There were several reports of Tn*7* derivatives located in bacterial chromosomes (Chen et al., 2018; Chen et al., 2019). To date, Tn*6726* and 40.7-kb Tn*7*-related element were the only two *bla*_NDM_-carrying Tn*7* derivatives integrated into chromosomes. Different to 40.7-kb Tn*7*-related element, Tn*6726* carried a series of backbone genes of IncHI3 plasmid, which means that *bla*_NDM_ together with its surrounding genetic environment in Tn*6726* might be originated from a IncHI3 plasmid. In summary, Tn*6512*-related ICEs, Tn*6523*-related IMEs, and Tn*7* derivatives recently began to be a reservoir of *bla*_NDM_ genes in Enterobacteriaceae.

Each of these six *bla*_NDM_-carrying genetic elements had large accessory resistance regions: i) Tn*6575*, Tn*6588*, Tn*6589*, and 40.7-kb Tn*7*-related element; each had a single MDR region, 85.0 kb, 90.1 kb, 66.4 kb, and 33.8 kb in length, respectively; ii) Tn*6726* contained a 162.6-kb IS*Kpn26*-composite transposon Tn*6728* integrated with a 40.9-kb MDR region; and iii) Tn*6576* harbored a 406.4-kb ICE Tn*6582* (containing two distinct MDR-1 and MDR-2 regions, 38.9 kb and 43.7 kb in length, respectively), and additionally a 55.2-kb IS*Ppu12*-composite Tn*6580b* that as a whole could be considered as a MDR region. Each of these large MDR regions had a very complex mosaic structure, which was composed of intact or residue mobile genetic elements including ISs, unit or composite transposons, integrons and putative resistance units, and likely assembled from complex transposition and homologous recombination.

**Figure 12:**
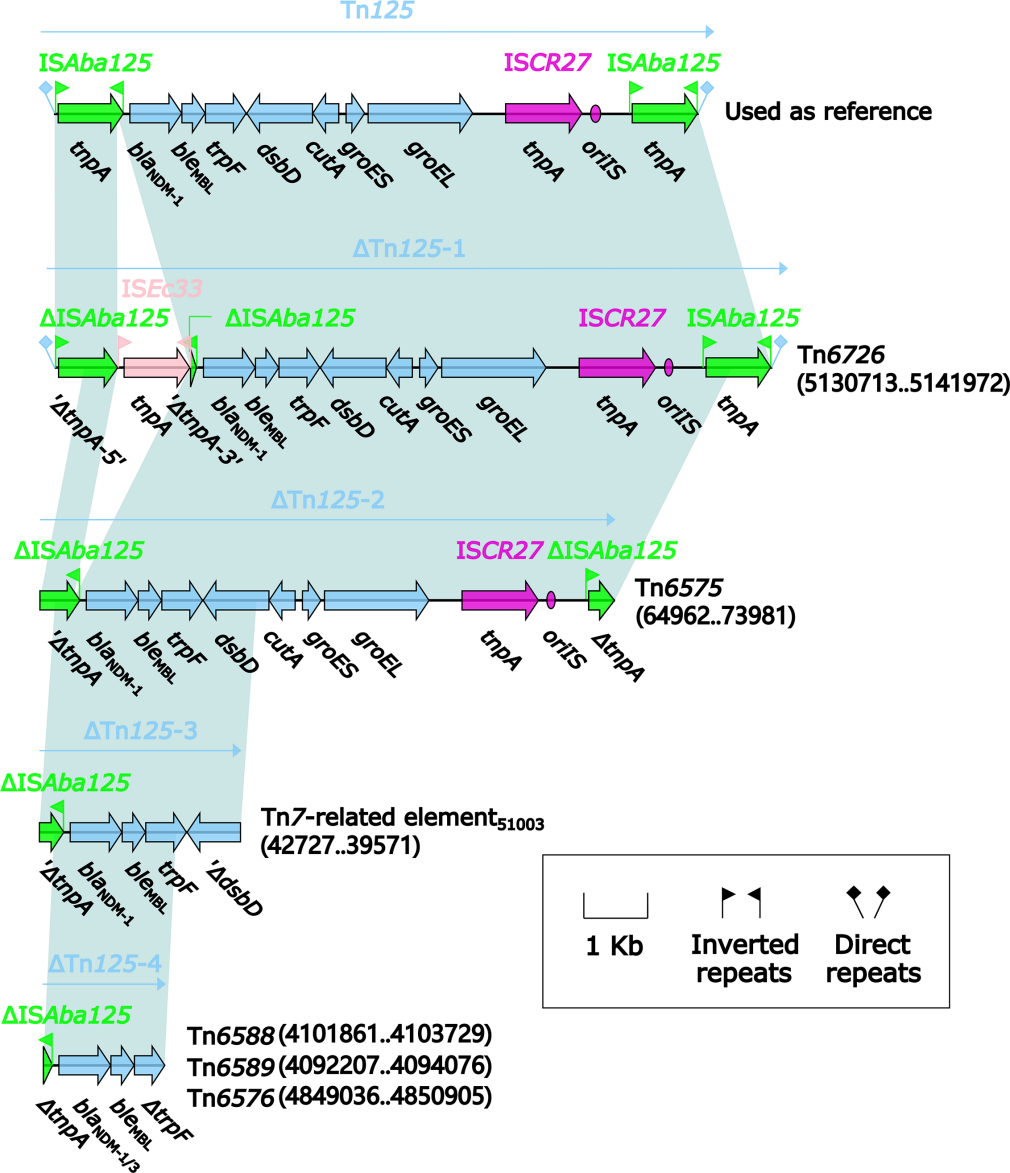
Comparison of Tn*125* and its four derivatives. Genes are denoted by arrows. Genes, mobile elements, and other features are colored based on their functional classification. Shading denotes regions of homology (nucleotide identity ≥95%). Numbers in brackets indicate nucleotide positions within Tn*6575* and the chromosomes of strains KP64, 51003, 1701091, 1701092, and QD23, respectively. The accession number of Tn*125* (Poirel et al., 2012) used as reference is JN872328.

Four different Tn*125* derivatives, namely ΔTn*125*-1, ΔTn*125*-2, ΔTn*125*-3, and ΔTn*125*-4 (**Figure 12**), were identified from the relevant MDR regions of these six *bla*_NDM_-carrying genetic elements. ΔTn*125*-1 from Tn*6726* and ΔTn*125*-2 from Tn*6575* highly resembled the prototype Tn*125*: ΔTn*125*-1 resulted from the insertion of IS*Ec33* into the left copy of IS*Aba125*, while terminal truncation of both copies of IS*Aba125* generated ΔTn*125*-2. It was thought that ΔTn*125*-1 and ΔTn*125*-2 were generated from transposition of Tn*125* into Tn*6726* and Tn*6575*, followed by further modular modifications such as insertion and truncation. ΔTn*125*-3 from 40.7-kb Tn*7*-related element and ΔTn*125*-4 from Tn*6588*, Tn*6589* and Tn*6576* had very short *bla*_NDM-1/-3_-carrying structures. ΔTn*125*-3 or ΔTn*125*-4 was captured by IS*CR1*, generating IS*CR1*–*ble*_MBL_–*bla*_NDM-1_ or IS*CR1*–*ble*_MBL_–*bla*_NDM-1/-3_–*arr-3* unit, respectively. Furthermore, the former unit was integrated into In2-16 (**Figure 10**) while the later one into In1247_Tn_ *_6588_* (**Figure 2**), In27_Tn_ *_6589_* (**Figure 3**) and In1021_Tn_ *_6576_* (**Figure 6**), manifesting as the VR2 regions of these integrons. Notably, two or more copies of *bla*_NDM-1/-3_ genes were found in each of Tn*6576*, Tn*6588*, Tn*6589*, and 40.7-kb Tn*7*-related element, which resulted from the presence of multiple ≥5.6-kb repeats (each harboring a Tn*125* derivative and the other components) in these four genetic elements.

Multiple copies of *bla*_NDM_ located in a single plasmid or chromosome were reported in previous studies (Jovcić et al., 2013; Shen et al., 2017; Feng et al., 2018), and all these *bla*_NDM_ genes were around IS*CR1*. Similarly, Tn*6576*, Tn*6588*, Tn*6589*, and 40.7-kb Tn*7*-related element in this study also contained IS*CR1*-around *bla*_NDM_ genes. It was confirmed that IS*CR1* captured adjacent genes (frequently including antibiotic resistance genes) at the end of its initiation of replication (*ori*IS) through rolling-circle transposition (Toleman et al., 2006). Our sequencing data suggested that IS*CR1* might experience multiple rounds of capturing *bla*_NDM_ and further integrating *bla*_NDM_ into the integrons, resulting in the presence of multiple copies of IS*CR1*-accociated *bla*_NDM_ genes in a single genetic element.

There were eight novel (firstly identified in this study) mobile genetic elements, including three ICEs Tn*6576*, Tn*6577*, and Tn*6582*, two IMEs Tn*6588* and Tn*6589*, two composite transposons Tn*6580a* and Tn*6580b*, and one integron In1718. Additional 12 genetic elements (IME: Tn*6590*; composite transposons: Tn*6578*, Tn*6581a*, Tn*6581b*, Tn*6581c*, and Tn*6728*; unit transposons: Tn*6726*, Tn*6727*, Tn*6909*, and Tn*6911*; IS: IS*Pvu1*; and 40.7-kb Tn*7*-related element) were newly designated (firstly designated in this study, but with previously determined sequences). The four previously designated ICEs/IMEs SGI1, R391, ICE*Pvu*ChnBC22, and GI*sul2* were renamed as standard Tn designations Tn*6523*, Tn*6512*, Tn*6575*, and Tn*6591*, respectively. All the putative resistance units presented in this work were annotated and collected in a custom and yet unpublished database.

## Conclusion

This study dealt with an extensive sequence comparison of 12 chromosomal genetic elements, including six *bla*_NDM_-carrying ones. All these *bla*_NDM_-carrying genetic elements had huge and complex MDR regions. The core *bla*_NDM_ genetic environments manifested as four different Tn*125* derivatives. Notably, two or more copies of *bla*_NDM_ were found in each of the four genetic elements. Egiht novel mobile elements were firstly identified, including three ICEs Tn*6576*, Tn*6577*, and Tn*6582*, two IMEs Tn*6588* and Tn*6589*, two composite transposons Tn*6580a* and Tn*6580b*, and one integron In1718. This study would provide a deeper genetic insight into the chromosomal integration of *bla*_NDM_-carrying genetic elements in Enterobacteriaceae.

## Data Availability Statement

The datasets generated for this study can be found in the complete chromosomal nucleotide sequences of 1701091, 1701092, QD23 and 51003, which were submitted to GenBank under accession numbers CP042860, CP042857, CP042858 and CP042861, respectively.

## Ethics Statement

This study uses the bacterial isolates obtained from the Chinese livestock farm and public hospitals as listed in **Table S1**. The local legislation did not require the study to be reviewed or approved by an ethics committee, because the bacterial isolates involved in this study was part of the routine laboratory procedures. The research involving biohazards and all related procedures were approved by the Biosafety Committee of the Beijing Institute of Microbiology and Epidemiology.

## Author Contributions

DZ and HY conceived the study and designed experimental procedures. XL, YJ, and FC performed the experiments. XL, XJ, and LZ analyzed the data. LH, DW and YS contributed to reagents and materials. XL and ZY wrote the original draft. DZ and HY reviewed the manuscript. All authors contributed to the article and approved the submitted version.

## Funding

This work was supported by the National Science and Technology Major Project (2018ZX10733402) of China, and the Foundation of the Public Welfare Program of Natural Science Foundation (LGF19H200006) of Zhejiang.

## Conflict of Interest

The authors declare that the research was conducted in the absence of any commercial or financial relationships that could be construed as a potential conflict of interest.
