# Supplementary material for: Chromosomal Integration of Huge and Complex bla NDM-Carrying Genetic Elements in Enterobacteriaceae
Source: Front Cell Infect Microbiol. 2021 Jun 15;11:690799. doi: 10.3389/fcimb.2021.690799 (PMC8239412; doi:10.3389/fcimb.2021.690799)
Supplement: Supplementary file 1 [file DataSheet_1.pdf]

Table S1. Major features of strains included in this study

Isolate	Bacterium	Country	Year	Specimen	Host	Source	Accession number
1701091	*Providencia rettgeri*	China	2017	Intestinal contents	Chicken	This study	CP042860
1701092	*Proteus mirabilis*	China	2017	Intestinal contents	Chicken	This study	CP042857
QD23	*Klebsiella pneumoniae*	China	2015	Urine	Homo sapiens	This study	CP042858
51003	*Providencia rettgeri*	China	2017	Urine	Homo sapiens	This study	CP042861
DT104	*Salmonella enterica* Typhimurium	Canada	2001	—	—	GenBank	AF261825
BC22	*Proteus vulgaris*	China	2018	Anal swab	Swine	GenBank	MH160822
107	*Providencia rettgeri*	United Kingdom	2002	—	—	GenBank	AY090559
KP64	*K. pneumoniae*	Thailand	2015	—	Homo sapiens	GenBank	AP018750
3.5-R3	*Escherichia coli*	Australia	2016	Feces	Homo sapiens	GenBank	KX117211
2457T	*Shigella flexneri*	Japan	1954	Feces	Homo sapiens	GenBank	AE014073

Table S2. Sub-regions and resistance genes in the genetic elements characterized in this work

Group	Genetic element	Sub-region harbored	Resistance marker	Resistance phenotype	Nucleotide position
Novel IME	Tn*6588*	In1718	*aacA4cr*	Fluoroquinolone and aminoglycoside resistance	4053214..4053813
			bla$_{OXA-1}$	β-lactam resistance	4053944..4054774
			catB3	Phenicol resistance	4054912..4055544
			arr-3	Rifampicin resistance	4055629..4056081
			qacED1	Quaternary ammonium compound resistance	4056304..4056651
			sul1	Sulphonamide resistance	4056645..4057484
		IS*26*–*mphE*–IS*26* unit	*mphE*	Macrolide resistance	4064893..4065777
			msrE	Macrolide, Lincosamide and Streptogramin B resistance	4065833..4067308
		In1247	*aadA2*	Aminoglycoside resistance	4108189..4108968
			lnuF2	Lincosamide	4107236..4108057
			ble$_{MBL}$	Bleomycin resistance	4077325..4077689<br>4083721..4084086<br>4090121..4090486<br>4096521..4096886<br>4102921..4103286
			bla$_{NDM-1}$	β-lactam resistance	4076509..4077321<br>4082905..4083717<br>4089305..4090117<br>4095705..4096517<br>4102105..4102917
			arr-3	Rifampicin resistance	4075316..4075768

					4081712..4082164<br>4088112..4088564<br>4094512..4094964<br>4100912..4101364
			aphA6	Aminoglycoside resistance	4070374..4071153
			qacED1	Quaternary ammonium compound resistance	4074746..4075093<br>4081143..4081489<br>4087542..4087889<br>4093942..4094289<br>4100342..4100689<br>4106741..4107088
			sul1	Sulphonamide resistance	4073913..4074752<br>4080312..4081149<br>4086709..4087548<br>4093109..4093948<br>4105908..4106747<br>4099509..4100348
		IS*CR2*–*floR* unit	*floR*	Phenicol resistance	4117563..4118777
		IS*CR2*–*sul2* unit	*sul2*	Sulphonamide resistance	4121280..4122095
		IS*Ec59*–*aph(4)-Ia*–*aacC4*–IS*26* unit	*aph(4)-Ia*	Aminoglycoside resistance	4126942..4127967
			aacC4	Aminoglycoside resistance	4128283..4129059
		ΔTn*4352*	*aphA1*	Aminoglycoside resistance	4129980..4130795
		Tn*6909*-related region	*dfrA12*	Trimethoprim resistance	4135903..4136400
			aadA2	Aminoglycoside resistance	4134704..4135483
Tn*6523*-related IMEs	Tn*6523*	In127	*tetA*(G)	Tetracycline resistance	32536..33663
			floR	Phenicol resistance	30337..31551
			aadA2	Aminoglycoside resistance	27853..28644
			qacED1	Quaternary ammonium	28808..29155

				compound resistance	38268..38615
			bla$_{\text{CARB-2}}$	β-lactam resistance	37185..38051
			sul1	Sulphonamide resistance	38609..39448
	Tn*6589*	In27	*dfrA12*	Trimethoprim resistance	4098798..4099295
			aadA2	Aminoglycoside resistance	4097599..4098378
			qacED1	Quaternary ammonium compound resistance	4078911..4079258<br>4090680..4091033<br>4097088..4097435
			sul1	Sulphonamide resistance	4078078..4078917<br>4084488..4085328<br>4089846..4090686<br>4096255..4097094
			ble$_{\text{MBL}}$	Bleomycin resistance	4081494..4081860<br>4093267..4093632
			bla$_{\text{NDM-1}}$	β-lactam resistance	4080674..4081490<br>4092451..4093263
			arr-3	Macrolide resistance	4079481..4079933<br>4091256..4091708
			qnrA1	Quinolone resistance	4075212..4075868<br>4086980..4087636
		IS*CR3*–*tetA*(G)–*floR* unit	*floR*	Phenicol resistance	4071158..4072372
			tetA(G)	Tetracycline resistance	4069046..4070221
		Tn*2*–*rmtB* region	*bla*$_{\text{TEM-1}}$	β-lactam resistance	4063556..4064416
			rmtB	Aminoglycoside resistance	4064586..4065341
		IS*CR2*–*floR* unit	*floR*	Phenicol resistance	4057365..4058579
		IS*Pa13*–*erm42*–IS*26* unit	*erm42*	Macrolide resistance	4050878..4051789
		aacC2–*tmrB* region	*aacC2*	Aminoglycoside resistance	4041098..4041958
			tmrB	Tunicamycin resistance	4041971..4042513

			*bla*TEM-1	β-lactam resistance	4046930..4047790
		In363	*dfrA1*	Trimethoprim resistance	4038243..4038716
			qacED1	Quaternary ammonium compound resistance	4037191..4037539
			sul1	Sulphonamide resistance	4036356..4037197
Tn*6512*-related ICEs	Tn*6512*	Tn*6578*	*aphA1*	Aminoglycoside resistance	12894..13709
		mer region	*mer* locus	Mercuric resistance	80391..83682
	Tn*6575*	Truncated IS*Ec59*–*aph(4)-Ia*–*aacC4*–IS*26* unit	*aph(4)-Ia*	Aminoglycoside resistance	12989..14014<br>44227..45252
			aacC4	Aminoglycoside resistance	14243..15019<br>43222..43998
		Unnamable In element	*aacA4cr*	Fluoroquinolone and aminoglycoside resistance	33722..34321<br>30630..31229
			*bla*OXA-1	β-lactam resistance	32761..33591
			catB3	Phenicol resistance	31991..32623
			arr-3	Rifampicin resistance	30081..30533<br>31454..31906
			ampC	β-lactam resistance	24780..25919
			qacED1	Quaternary ammonium compound resistance	29511..29858
			sul1	Sulphonamide resistance	22364..23203<br>28678..29517
		Tn*4352*	*aphA1*	Aminoglycoside resistance	35254..36069
		IS*26*–*cfr*–IS*26* unit	*cfr*	Phenicol resistance	37586..38635
		Tn*6581a*	*bleO*	Bleomycin resistance	41824..42210
		In525	*aadA2*	Aminoglycoside resistance	54081..54554
			ereA	Macrolide resistance	54747..55967
			dfrA32	Trimethoprim resistance	54081..54554

		ΔTn*6309*	*tetA*(C)	Tetracycline resistance	61430..62620
		ΔTn*125*-2	*bla*$_{NDM-1}$	β-lactam resistance	65690..66502
			ble$_{MBL}$	Bleomycin resistance	66506..66871
		Tn*6911*-related region	*qacED1*	Quaternary ammonium compound resistance	75746..76093
			sul1	Sulphonamide resistance	74913..75752
		Truncated IS*CR2*–*floR* unit	*floR*	Phenicol resistance	84749..85963
		Truncated IS*CR2*–*sul2* unit	*sul2*	Sulphonamide resistance	88619..89434
		ΔTn*5393c*	*strA*	Aminoglycoside resistance	87755..88558
			strB	Aminoglycoside resistance	86919..87755
	Tn*6576*	In525	*aadA2*	Aminoglycoside resistance	4512080..4512859
			ereA	Macrolide resistance	4512956..4514176
			dfrA32	Trimethoprim resistance	4514369..4514842
		Truncated *chrA*–*orf98* unit	*chrA*	Chromate resistance	4516708..4517913
		Tn*6581b*	*bleO*	Bleomycin resistance	4519258..4519644<br>4625360..4625746
		Unnamable In37-like element	*aacA4cr*	Fluoroquinolone and aminoglycoside resistance	4521355..4521954<br>4625746..4628056
			bla$_{OXA-1}$	β-lactam resistance	4522085..4522915<br>4628187..4629017
			catB3	Phenicol resistance	4523053..4523685;<br>4629155..4629787
			arr-3	Rifampicin resistance	4523770..4524222<br>4629872..4630324
			qacED1	Quaternary ammonium compound resistance	4524445..4524792<br>4630547..4630894
			sul1	Sulphonamide resistance	4524786..4525625<br>4630888..4631727

		IS*Ec59*–*aph(4)-Ia* –*aacC4*–IS*26* unit	*aph(4)-Ia*	Aminoglycoside resistance	4526607..4527383
			aacC4	Aminoglycoside resistance	4527612..4528637
		IS*CR2*–*sul2* unit	*sul2*	Sulphonamide resistance	4533484..4534299
		Truncated IS*CR2*–*floR* unit	*floR*	Phenicol resistance	4536802..4538016
		ΔTn*6503a*	*bla*$_{\text{CTX-M-14}}$	β-lactam resistance	4547007..4547882
		IS*26*–*fosA3*–IS*26* unit	*fosA3*	Fosfomycin resistance	4551726..4552142
		Tn*4352*	*aphA1*	Aminoglycoside resistance	4554787..4555602<br>4616494..4617309<br>4634951..4635766
		ΔTn*6309*	*tetA*(C)	Tetracycline resistance	4558081..4559271
		ΔTn*6029*	*sul2*	Sulphonamide resistance	4610858..4611673
			strA	Aminoglycoside resistance	4611734..4612537
			strB	Aminoglycoside resistance	4612537..4613373
		Truncated *aacC2*–*tmrB* region	*aacC2*	Aminoglycoside resistance	4614371..4615231<br>4632828..4633688<br>4859983..4860855<br>4881427..4882287
		IS*26*–*mph*(A)–IS*6100* unit	*mph*(A)	Macrolide resistance	4618286..4619191<br>4636743..4637648<br>4840469..4841374<br>4861920..4862830
		chrA–*orf98* unit	*chrA*	Chromate resistance	4622810..4624015<br>4641267..4642472<br>4844992..4846197<br>4866450..4867655
		In27	*dfrA12*	Trimethoprim resistance	4645502..4645999
			aadA2	Aminoglycoside resistance	4644303..4645082
			qacED1	Quaternary ammonium	4643792..4644139

				compound resistance	
			sul1	Sulphonamide resistance	4642959..4643798
		sil–cop region	*sil* locus	Silver resistance	4696846..4709292
			cop locus	Copper resistance	4710590..4718123
		ΔTn*21*	*mer* locus	Mercuric resistance	4828474..4832436
		aacC2–tmrB region	*aacC2*	Aminoglycoside resistance	4838550..4839410
			tmrB	Tunicamycin resistance	4837995..4838537
			$bla_{\text{TEM-1}}$	β-lactam resistance	4832718..4833578
		In1021	*aacA4cr*	Fluoroquinolone and aminoglycoside resistance	4856564..4857164<br>4878020..4878619
			dfrA27	Trimethoprim resistance	4855407..4855880<br>4876865..4877338
			aadA16	Aminoglycoside resistance	4854381..4855226<br>4875839..4876684
			ble_{MBL}	Bleomycin resistance	4850096..4850461<br>4871554..4871919
			$bla_{\text{NDM-3}}$	β-lactam resistance	4849280..4850092<br>4870738..4871550
			arr-3	Rifampicin resistance	4848087..4848539<br>4856014..4856467<br>4869545..4869997<br>4877471..4877923
			qacED1	Quaternary ammonium compound resistance	4847517..4847864<br>4853917..4854264<br>4868975..4869322<br>4875375..4875722
			sul1	Sulphonamide resistance	4846684..4847523<br>4853084..4853923

					4868142..4868981<br>4874542..4875381
		ΔTn*5393c*	*strA*	Aminoglycoside resistance	5003486..5004289
			strB	Aminoglycoside resistance	5004289..5005125
		IS*CR2 –floR* unit	*floR*	Phenicol resistance	5006081..5007295
		Truncated IS*CR2–sul2* unit	*sul2*	Sulphonamide resistance	5002610..5003425
	Tn*6577*	In525	*aadA2*	Aminoglycoside resistance	3266082..3266861
			ereA	Macrolide resistance	3266958..3268178
			dfrA32	Trimethoprim resistance	3268371..3268844
		Truncated *chrA–orf98* unit	*chrA*	Chromate resistance	3270710..3271915
		Tn*6581c*	*bleO*	Bleomycin resistance	3273260..3273646
		Unnamable In37-like element	*aacA4cr*	Fluoroquinolone and aminoglycoside resistance	3279093..3279692
			bla_{OXA-1}	β-lactam resistance	3278132..3278962
			catB3	Phenicol resistance	3277362..3277994
			arr-3	Rifampicin resistance	3276825..3277277
			qacED1	Quaternary ammonium compound resistance	3276255..3276602
			sul1	Sulphonamide resistance	3275422..3276261
		IS*26–aacC4*–IS*Ec59–aph(4)-Ia* unit	*aph(4)-Ia*	Aminoglycoside resistance	3281614..3282639
			aacC4	Aminoglycoside resistance	3280609..3281385
		IS*CR2–sul2* unit	*sul2*	Sulphonamide resistance	3287486..3288301<br>3364494..3365309
		Truncated IS*CR2–floR* unit	*floR*	Phenicol resistance	3290804..3292018
		ΔTn*6503a*	$bla_{CTX-M-14}$	β-lactam resistance	3301009..3301884
		ΔTn*6309*	*tetA*(C)	Tetracycline resistance	3306927..3308117
		-	bla_{HMS-1}	β-lactam resistance	3360360..3361223
Tn*7*-related	Tn*7*	In2-4	*aadA1*	Aminoglycoside resistance	9884..10672

elements			*sat2*	Aminoglycoside resistance	10730..11254
			dfrA1	Trimethoprim resistance	11349..11822
	Tn*6726*	In2-3	*sat2*	Aminoglycoside resistance	5062468..5062992
			dfrA1	Trimethoprim resistance	5063087..5063560
		ΔTn*1548*	*dfrA12*	Trimethoprim resistance	5112659..5113156
			aadA2	Aminoglycoside resistance	5113576..5114355
			qacED1	Quaternary ammonium compound resistance	5114519..5114866
			sul1	Sulphonamide resistance	5114860..5115699
			armA	Aminoglycoside resistance	5119044..5119817
			msr(E)	Macrolide resistance	5122116..5123591
			mph(E)	Macrolide resistance	5123647..5124531
		Truncated IS*Aba14*–*aphA6*–IS*Aba14* unit	*aphA6*	Aminoglycoside resistance	5129839..5130618
		ΔTn*125*-1	bla_{NDM-1}	β-lactam resistance	5133051..5133863
			ble_{MBL}	Bleomycin resistance	5133867..5134232
	40.7-kb Tn7-related element	In2-16	*lnu*(F)	Lincosamide resistance	33793..34638
			dfrA1	Trimethoprim resistance	34635..35108
			aadA1a	Aminoglycoside resistance	35125..35916
			bla_{NDM-1}	β-lactam resistance	41431..42243<br>47085..47897
			ble_{MBL}	Bleomycin resistance	41062..41427<br>46716..47081
			qacED1	Quaternary ammonium compound resistance	36214..36561
			sul1	Sulphonamide resistance	36555..37394
		ΔTn*1548*	*armA*	Aminoglycoside resistance	52047..52820
			msr(E)	Macrolide resistance	55119..56594

			mph(E)	Macrolide resistance	56650..57534
		ΔTn*4352*	*aphA1*	Aminoglycoside resistance	62707..63522
Tn*6591*-related IMEs	Tn*6591*	Backbone	*ars* locus	Arsenic resistance	2603040..2605703
		IS*CR2*–*sul2* unit	*sul2*	Sulphonamide resistance	2598850..2599665
	Tn*6590*	Backbone	*ars* locus	Arsenic resistance	2261006..2263669
		Truncated IS*CR2*–*sul2* unit	*sul2*	Sulphonamide resistance	2267197..2268012
		ΔTn*5393c*	*strA*	Aminoglycoside resistance	2266333..2267136
			strB	Aminoglycoside resistance	2265497..2266333
