# Supplementary material for: Chromosomal Integration of Huge and Complex bla NDM-Carrying Genetic Elements in Enterobacteriaceae
Source: Front Cell Infect Microbiol. 2021 Jun 15;11:690799. doi: 10.3389/fcimb.2021.690799 (PMC8239412; doi:10.3389/fcimb.2021.690799)
Supplement: Supplementary file 2 [file Image_1.pdf]

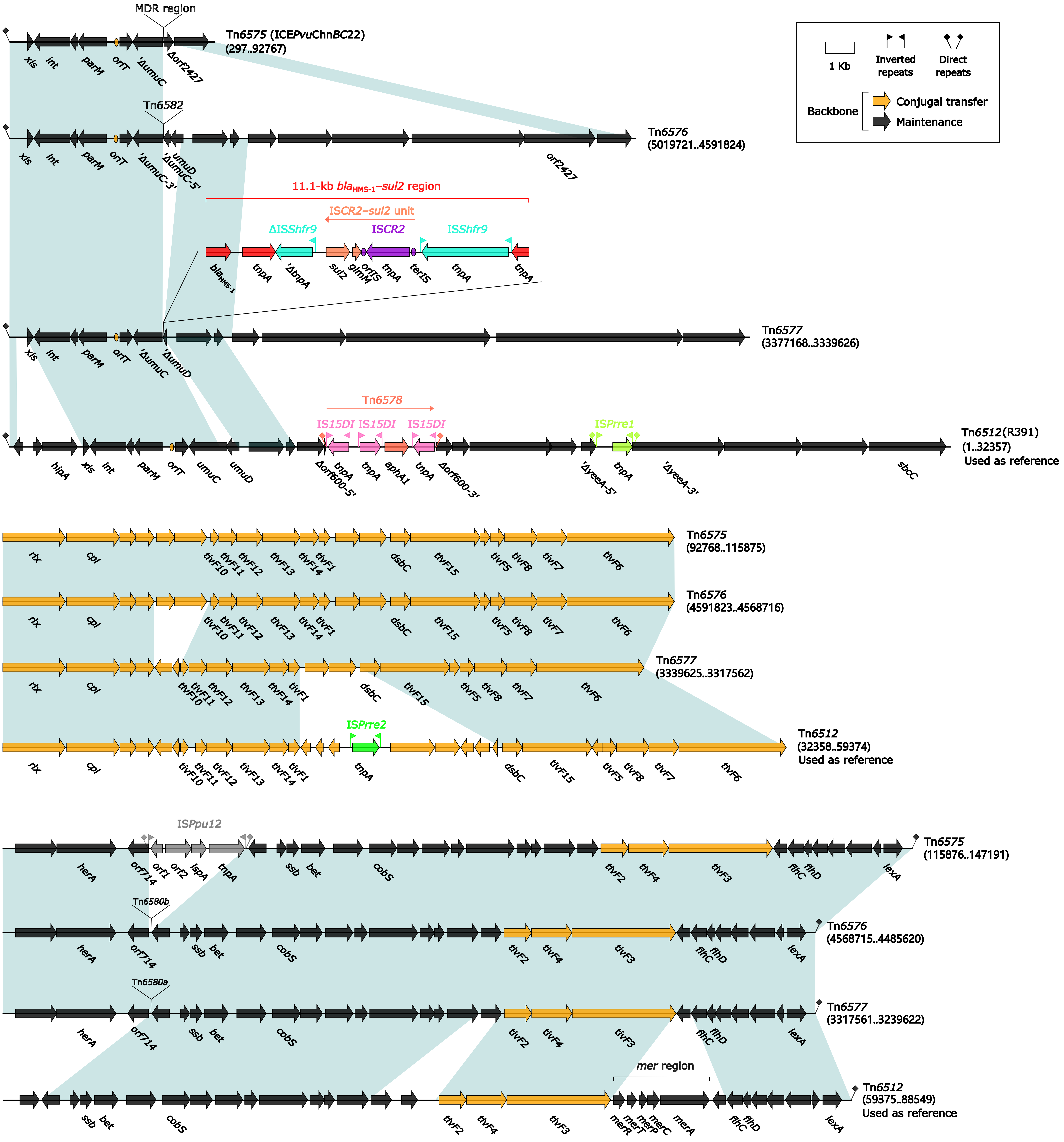

Figure S1. Comparison of four related ICEs Tn*6512*, Tn*6575*, Tn*6576*, and Tn*6577*. Genes are denoted by arrows. Genes, mobile elements and other features are colored based on their functional classification. Shading denotes regions of homology (nucleotide identity ≥95%). Numbers in brackets indicate nucleotide positions within Tn*6512*,[1] Tn*6575*, and the chromosomes of strains QD23 and 1701092, respectively. The accession number of Tn*6512* used as reference is AY090559.

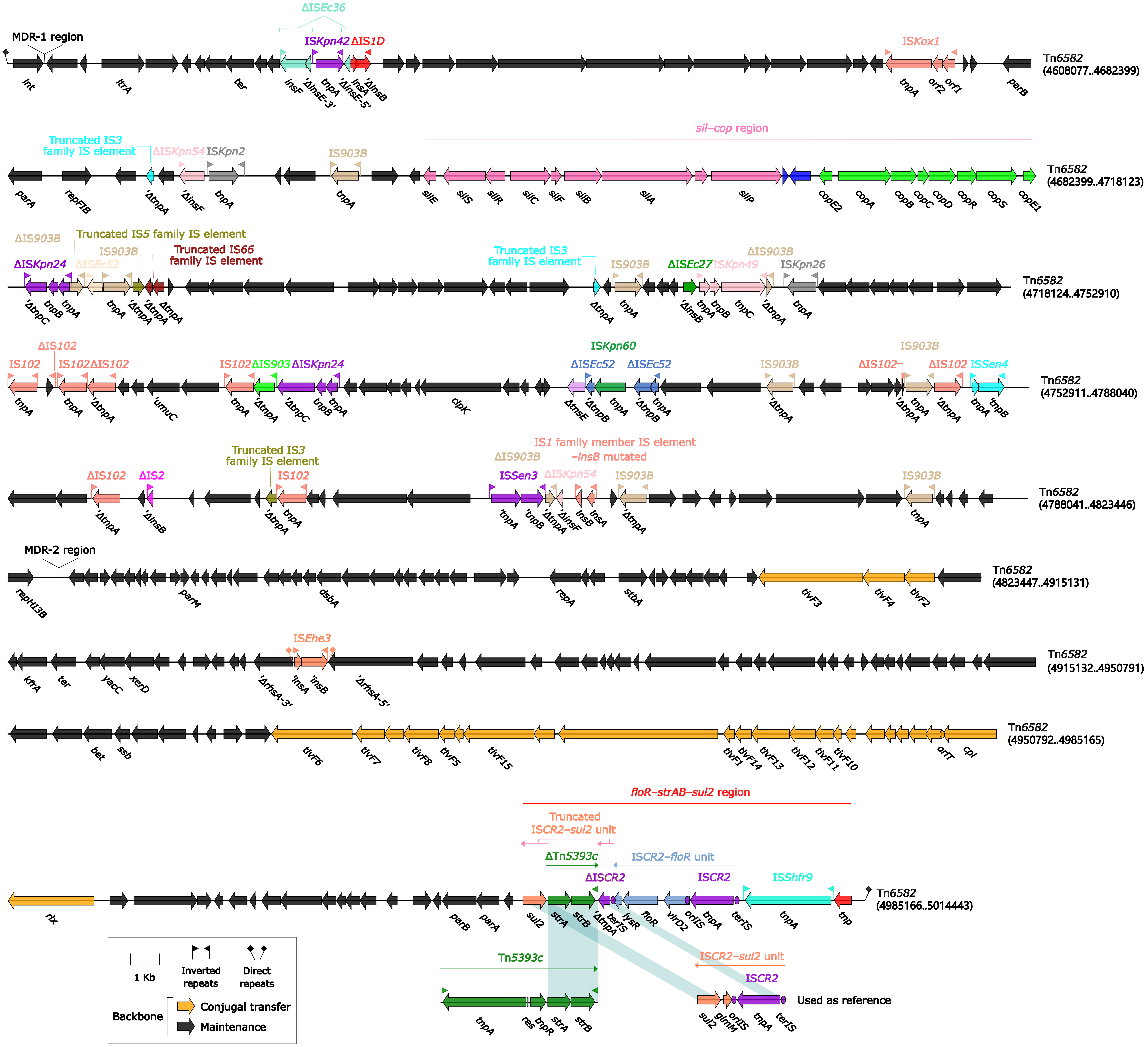

Figure S2. Organization of Tn*6582*, and comparison to related regions. Genes are denoted by arrows. Genes, mobile elements and other features are colored based on their functional classification. Shading denotes regions of homology (nucleotide identity ≥95%). Numbers in brackets indicate nucleotide positions within the chromosome of strain QD23. The accession numbers of Tn*5393c*[2] and IS*CR2*–*sul2* unit used as reference are AF262622 and AE014073, respectively.

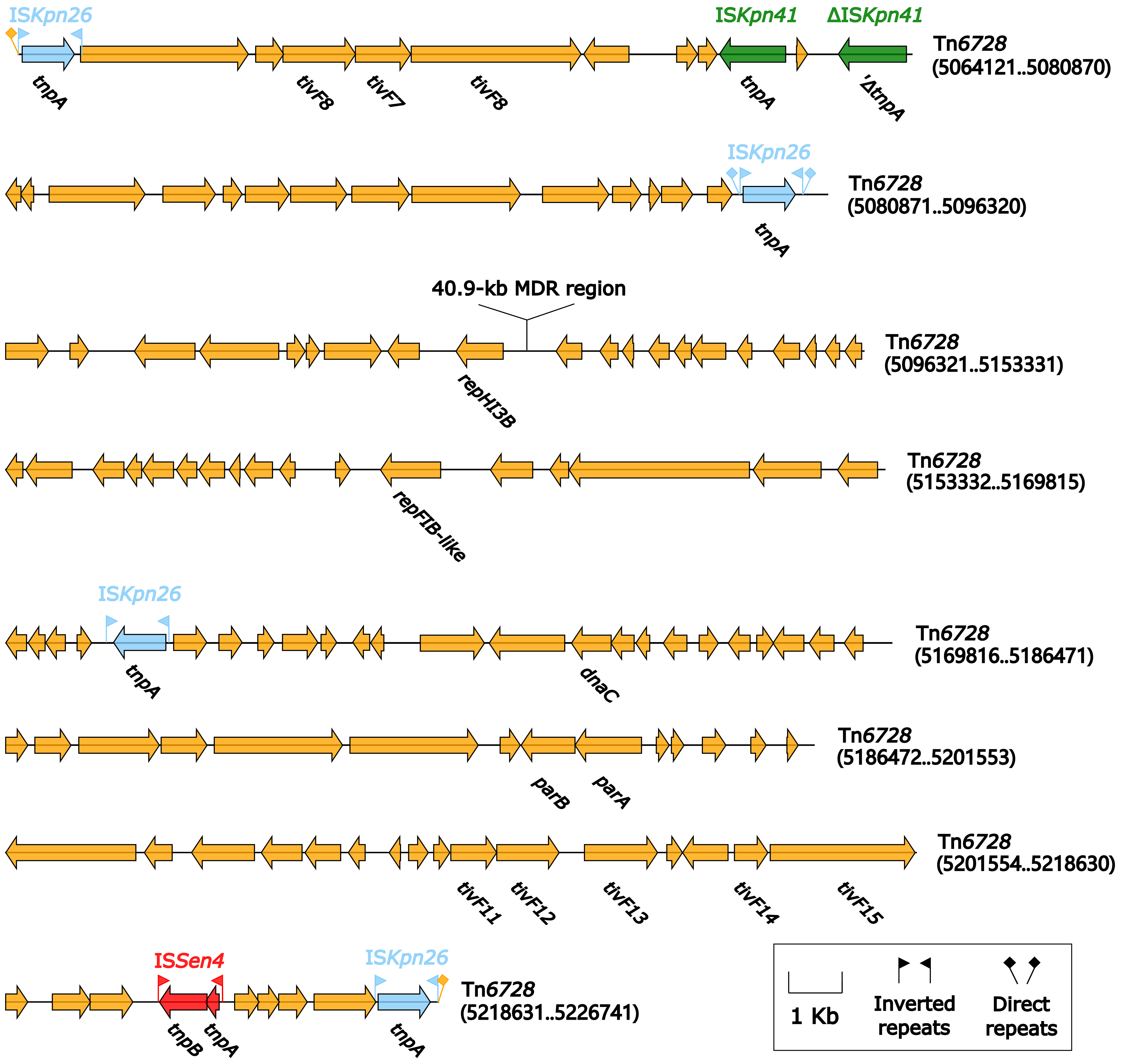

Figure S3. Organization of Tn*6728*. Genes are denoted by arrows. Genes, mobile elements and other features are colored based on their functional classification. Numbers in brackets indicate nucleotide positions within the chromosome of strain KP64.

References

1 Boltner D, MacMahon C, Pembroke JT et al. R391: a conjugative integrating mosaic comprised of phage, plasmid, and transposon elements. J Bacteriol 2002; **184**: 5158-69.

2 L'Abée-Lund TM, Sørum H. Functional Tn*5393*-like transposon in the R plasmid pRAS2 from the fish pathogen *Aeromonas salmonicida* subspecies *salmonicida* isolated in Norway. Appl Environ Microbiol 2000; **66**: 5533-5.
